# Designing a set of reference standards for non-targeted analysis of polymer additives extracted from medical devices

**DOI:** 10.1038/s41370-025-00788-w

**Published:** 2025-07-09

**Authors:** Byeong Hwa Yun, Amali Herath, Ying Jin, Jamie Kim, Kerry Belton, Echoleah Rufer, Omar Rivera Betancourt

**Affiliations:** 1 Division of Biology, Chemistry and Materials Science (DBCMS), Office of Science and Engineering Laboratories (OSEL), Center for Devices and Radiological Health (CDRH), U.S. Food and Drug Administration, Silver Spring, MD 20993 USA; 2 Division of Non-Clinical Science (DNCS), Office of Science (OS), Center for Tobacco Products (CTP), U.S. Food and Drug Administration, Silver Spring, MD 20993 USA

**Keywords:** Extractables, Relative response factor, Medical device, Biocompatibility, Mass spectrometry

## Abstract

**Background:**

The accurate analysis of extractables and leachables (E&L) from medical devices is crucial for the reliable safety risk assessment of substances to which patients and users may be exposed. The extractable profile of medical devices is often complex and unpredictable, thus improper selection of reference standards can lead to irreproducible chemical analyses between laboratories. ISO 10993-18, the international consensus standard for chemical characterization of medical devices, does not specify a process for selection of appropriate chemical reference standards for non-targeted analysis of E&L, leading to a variety of approaches being used.

**Objective:**

This study seeks to set out requirements for building a comprehensive list of chemical reference standards for non-targeted analysis of E&L and propose suggestions for selecting appropriate standards to enhance the consistency of chemical analysis.

**Methods:**

Criteria for selecting reference standards for non-targeted analysis of E&L in medical devices were developed using relevant polymer additives as a model system. The Relative Response Factor (RRF) values of the selected reference standards were determined using GC-MS and LC-MS analysis across three different concentrations. A system was developed to rank the toxicological hazards of the selected reference standards.

**Results:**

A list of 106 reference standards of polymer additives was compiled, encompassing a wide range of physicochemical properties and broad toxicological coverage. Statistical analyses of these chemicals revealed there was no significant correlation between their six physicochemical properties and the corresponding relative response factors measured by GC-MS and LC-MS techniques.

**Impact:**

Accurate chemical identification and quantification of extractable substances from medical devices is important for chemical characterization of medical devices. The accurate quantitation of extractable chemicals in medical devices through non-targeted analysis is dependent on the proper selection of reference standards. We have proposed a set of reference standards intended to enhance the confidence in quantitation of device extractables, covering a broad range of structural and physicochemical diversity. This set of reference standards may assist chemistry laboratories in developing robust screening methods for extractables in medical devices, supporting the accurate characterization of medical devices.

## Introduction

The U.S. FDA’s Center for Devices and Radiological Health (CDRH) has observed a significant increase in pre-market medical device submissions over the past three years. Recently, from 2021 to 2023, over 19,000 submissions were received each year, and this trend is expected to continue [1–3]. FDA’s biocompatibility guidance, *Use of International Standard ISO 10993-1, “Biological evaluation of medical devices - Part 1: Evaluation and testing within a risk management process”*, outlines methods to evaluate the biological safety of a medical device that may come into contact with the human body [4]. ISO 10993, part18 published in 2020, defines chemical characterization as the process of acquiring chemical information through both data collection, such as literature reviews, and data generation using chemical analysis [5]. Importantly, chemical characterization of medical device extracts can be used in lieu of certain biocompatibility tests that require extended testing times or the use of animals [4, 6]. The identification and quantification of extractable and leachable constituents are primarily conducted to support toxicological risk assessments and are often used in the biocompatibility evaluation of medical devices. For medical devices, extractables refer to substances inherent in a material, component, or system, capable of being extracted by a solvent while leachables are substances that transfer from the medical device to the user during normal use [7, 8].

Chemical analysis of medical devices aims to provide a comprehensive identification and quantitation of their E&L constituents above the analytical reporting limit. This information directly supports the device’s toxicological risk assessment by identifying substances to which a patient may be exposed, and estimating the extent of potential exposure based on the quantity of those identified substances. The inherent unpredictability and complexity of extractable profiles in medical devices present a challenge in measuring the quantities of unknown extractables using targeted analysis with established internal standards. Therefore, non-targeted analysis (NTA) is frequently employed to characterize the extractable chemicals in the medical devices. Unlike targeted analysis, which focuses on specific compounds with known standards, the NTA uses a set of reference standard materials (RM) to identify broader classes or groups of compounds within the sample.

In general, the primary source of device extractables are the materials used in medical device construction and chemicals introduced during the manufacturing process (e.g., detergents, mold release agents, transfer from manufacturing equipment). In practice, polymeric materials are the primary source of E&L in medical devices. These materials contain homologous polymers combined with additives to enhance the properties of the polymer for the intended use and manufacturing needs. Given the wide variety of constituents in polymeric materials, we designed a practical experimental approach by classifying the potential extractables into three categories based on the challenges of analytical method development using mass spectrometry; (Tier I) polymer additives added to polymer materials, (Tier II) unreacted monomers or short-chain polymers from degradation or uncompleted polymerization reaction, and (Tier III) colorants and pigments added to polymeric materials. In this study, we focused on the chemical analysis of the Tier I chemicals to develop a set of RM for profiling extractables in polymeric medical device material. Future studies will assess candidates for Tiers II and III and explore potential expansions of Tier I.

To develop a practical experimental approach for the Tier I chemicals, it is essential to establish appropriate selection benchmarks for choosing reference standards, which is important for accurate quantitation of extractables using NTA methods. The following six points were established to prioritize the selection of polymer additives for this study:

1. *Reference standard availability*: the candidate reference standards are expected to be widely commercially available,
2. *Chemical relevance to expected analytes*: the chemical classes of reference standards are expected to be align with the anticipated E&L profile in a sample device, thereby enhancing confidence in their identification,
3. *Chemical Stability*: the selected reference standards need to be stable and readily detectable during analysis using gas chromatography-mass spectrometry (GC-MS) and/or liquid chromatography-mass spectrometry (LC-MS) techniques,
4. *Toxicological coverage*: chemicals with toxicological hazards will be included in the list of RM. Although the RM list is a surrogate for actual extractables, including potential extractables with diverse toxicological profiles offers additional evidence that substances with a range of toxicological characteristics are adequately covered. This is important because the E&L analysis is primarily leveraged to determine toxicological risk. Additionally, it is desirable to minimize highly toxic chemicals to reduce occupational safety risk for laboratory personnel during E&L testing,
5. *Frequency of use*: if a chemical has the potential for cumulative exposure due to applications beyond being a polymer additive, the likelihood of detecting this chemical in human is higher compared to chemicals with more limited applications. Additionally, it may be of greater interest from a toxicological perspective,
6. *Analytical compatibility*: the physicochemical properties of RM are expected to fall within the detectable and separatable range of GC-MS and LC-MS techniques selected for this study. By establishing a comprehensive list of E&L using GC-MS and LC-MS, we can delineate the detectable E&L area in a chemical coverage map.

For E&L profiling using NTA methods, GC-MS and LC-MS are the analytical techniques most commonly employed. These methods can identify and quantify a broad range of E&L chemicals across a wide spectrum of polarities [5, 9, 10]. GC-MS method, which have been thoroughly developed over the past few decades, is widely regarded for its high reproducibility in detecting volatile organic compounds. Because of the high level of instrument standardization and reliability over extended periods, data collected by different laboratories can be directly compared providing highly accurate and reproducible test results [11]. LC-MS technique, which provide softer ionization conditions than electron ionization (EI) in GC-MS, detect a wider range of analytes (i.e., semi-volatile, non-volatile, thermally labile, or polar organic compounds). If the analytes are dissolved in the mobile phase, LC-MS can analyze even the least volatile or thermally unstable compounds that are difficult to assess using GC-MS. This is a notable advantage when analyzing samples of unknown composition [12].

To characterize the E&L profile of a medical device, the first step involves extracting substances from devices using solvents. Employing solvents with varying polarities facilitates the extraction of a diverse array of E&L compounds.

ISO 10993-18:2020 introduces the analytical evaluation threshold (AET), which defines the concentration above which E&L should be identified and quantified. The AET concept addresses the challenge of characterizing numerous unknown chemicals identified through NTA, particularly those present at low concentrations and unlikely to pose toxicological concern at their potential exposure levels. Therefore, the AET streamlines the evaluation process by focusing resources on higher-concentration (and possibly higher toxicological risk) chemicals [5, 13]. Applying an appropriate AET is crucial for conducting a robust toxicological risk assessment of E&L compounds and ensuring patient safety [14, 15]. The formula for calculating the AET is provided in the supplementary information of this study (Equation S1) as described in Clause E.2 of ISO 10993-18:2020 [5]. Among the parameters used to calculate the AET, the uncertainty factor (UF) addresses the analytical uncertainty associated with screening methods employed to estimate the quantity of extractables in the device extract. The UF is analytical technique-specific [15] and calculated using the following formula:

$${UF}\;{=}\;\frac{1}{\left(1-{RSD}\right)}$$

Where RSD is the relative standard deviation of the response factors from the reference standard database [5].

The selection of reference standards significantly impacts the distribution of response factors, which in turn critically affects the calculated UF [16, 17]. When selecting reference standards for E&L analysis, chemical testing laboratories may need to consider several factors, including the availability of standard chemicals, the number of the selected RMs, the distribution of their relative responses to the internal standard, and their relevance to the expected chemical classes of E&L. The selection of reference standard is important because an inappropriate selection of RMs can result in an underestimation of the UF and lead an overestimating the AET. Consequently, a significant portion of the E&L profile may be underreported in the toxicological risk assessment [18]. This is concerning because it could lead to an underestimation of a device’s toxicological risk to a patient and an inaccurate safety evaluation.

The RSD in the above UF calculation equation can be determined from the variance of relative response factors (RRF) within a selected set of reference standards when a direct comparison with a known internal standard is not feasible due to the unpredictable extractable profile. A quantitative approach for calculating the UF using RRF is described in Clause E.3 “Determination of the uncertainty factor” of ISO 10993-18:2020. The RRF of an extractable can be calculated by the ratio of the signal intensity of the compound over that of a reference standard. The RRF is a correction factor that ensures consistent quantitation by accounting for variations in signal response between the analyte and reference standard.

Ultimately, chemical analysis data is used to assess potential safety risk using toxicological risk assessment. A chemical’s toxicity can be partially determined by physicochemical parameters. As an example, a large molecular weight substance might not be able to cross cell membranes thereby preventing access to cellular components where it could cause a toxic effect. If the set of reference standards excludes a certain chemical space, such as low molecular weight substances, it might also miss certain toxicological profiles. To evaluate toxicological coverage of the set of RM, we developed a toxicological hazard ranking system to address specific needs for medical devices. This system aims to provide a standardized approach for ranking and comparing individual toxicological endpoints and a chemical’s overall hazard. Our ranking system was built using established frameworks such as Clean Production Action’s GreenScreen® for Safer Chemicals, the Globally Harmonized System (GHS) for hazard classification, and U.S. EPA Cheminformatics [19–21]. A broad toxicological ranking of reference standards within the NTA further validates its suitability for comprehensive risk assessments.

Finally, this study suggests a set of reference standard chemical materials focused on polymer additives and provides their RRF values measured by GC-MS and LC-MS across three different concentrations for quantitation of extractables in medical devices employing NTA technique. This set comprises one hundred and six chemicals chosen from the chemical analysis reports of medical devices submitted to the FDA between 2016 to 2023, and feedback from internal and external stakeholders. We further explored how certain physicochemical properties of the RMs affect the signal intensity of individual chemicals through GC-MS and LC-MS. The selected six physicochemical properties can be utilized to predict chromatographic behavior, mass spectrometric performance, and chemical purity. These properties include molecular weight, double bond equivalents, boiling point, pKa, log P, and refractive index (RI). This set of RM contributes to developing a chemical coverage map for analyzing medical device E&L that is intended to assist chemistry laboratories in ensuring the robustness of chemical characterization methods used to assess medical device safety.

## Materials and methods

### Materials

Chemicals were purchased from various suppliers and the entire list of vendors for each chemical is shown in Table S4. The LC-MS grade organic and aqueous solvents were used for sample preparation and LC-MS analysis, including methanol (Honeywell, Charlotte, NC USA), acetonitrile, and water. Specifically, hexane (ThermoFisher Scientific, Waltham, MA USA) was used to dissolve a couple of GC-MS samples that were insoluble in methanol. The diluted stock solutions and working samples for GC-MS and LC-MS analyses were prepared in 2 mL Crimp Top vials with crimp cap (Agilent, Santa Clara, CA USA) or 2 mL vials with 9 mm screw cap of PTFE/Silicone septa (ThermoFischer Scientific, Waltham, MA). Positive ion and negative ion calibration solutions for Thermo Q Exactive system were purchased from ThermoFisher Scientific. The chemical structures of 106 chemicals are shown in Supplementary Information (Fig. S1) and their physicochemical properties are included in Table S1. In the preliminary phase of study, we initially chose 32 chemicals (identified by codes from RM1 to RM32 in Table S1) based on a priori knowledge, anticipating strong or medium responses in GC-MS and LC-MS. This list of selected chemicals was reviewed by FDA chemists and external medical device testing laboratories. Later, the methods were validated with additional chemicals with medium to weak signal response in GC-MS and LC-MS (identified by codes from RM33 to RM106 in Table S1).

### Sample preparation

Individual stock solutions of reference chemicals were prepared at concentrations of 1, 0.2, or 0.1 mg/mL in methanol, based on their solubilities. This solutions were stored in 2 mL amber glass vials with caps at −80 °C. Samples insoluble or less soluble in methanol were dissolved in acetonitrile or hexane. For working solution preparation, the stock solutions stored at −80 °C were thawed, sonicated in a water bath at room temperature, and briefly spun down before further dilution. Working solutions were prepared at concentrations of 5, 10, and 20 µg/mL. For both GC-MS and LC-MS analyses, the samples were grouped by 3 to 10 chemicals including the respective internal standard to streamline the sample analysis process. When the reference chemicals were grouped, the chemicals were meticulously based on solvent compatibility and estimated elution time to augment the chromatographic resolution of sample peaks. This approach ensured optimal performance by aligning the chemical properties with the chromatographic conditions specific to each mixture. Table S13 provides a comprehensive list of the subsets and organic solvents employed for sample preparation. All working solutions were freshly prepared from the stock solution before analysis.

For GC-MS analysis, each stock solution was serially diluted to prepare three replicate samples. RM5 (2,4-ditert-butylphenol) was employed as the internal standard for GC-MS analysis. For LC-MS analysis, the samples were freshly prepared on three different days in triplicate, to evaluate the reproducibility of signal intensities. RM81 (trihexyl 2-butanoyloxypropane-1,2,3-tricarboxylate) was employed as an internal standard for positive ion detection mode, while RM92 ((2,4-dihydroxyphenyl)-phenylmethanone) was employed for negative ion detection mode in LC-MS analyses. The type of analytical method for each chemical was determined based on an a priori mass spectra database. If the mass spectrum of an analyte was unavailable in database, we considered the chemical’s physicochemical properties, along with feedback from FDA reviewers and external test laboratories to determine appropriate analytical method.

### GC-MS analysis

Agilent Technologies 6890 N gas chromatograph equipped with a 5975B mass selective detector (Santa Clara, CA USA) was used for GC-MS analysis. All samples were assessed using an Agilent DB-5HT (30 m × 0.25 mm i.d., 0.1 µm) or Agilent HP-5MS (30 m × 0.25 mm i.d., 0.25 µm) capillary column. The column oven temperature was set to 40 °C and increased to either 310 °C or 340 °C at heating rates of 15 °C/min or 7.5 °C/min, respectively, over a duration of 40 min. A single taper liner (4 mm i.d.) was used and the inlet temperature was set to 310 °C. A splitless injection mode was used throughout the study, with a consistent sample injection volume of 1 µL for the entire GC-MS analysis. Ultra-high purity Helium (99.999%) was used as carrier gas at a flow rate of 1 mL/min. The transfer line temperature was set to either 300 °C or 325 °C. The mass scan range was *m/z* 35 - 650 and EI was performed at 70 eV. GC-MS experimental data were analyzed using Agilent MassHunter Qualitative Analysis software.

The performance evaluation of GC-MS method was conducted by assessing the within-day repeatability and the between-day reproducibility of four representative reference standards (RM5, RM33, RM52, and RM66), analyzed in triplicate over three different days. These samples were prepared at three concentration levels: 5, 10, and 20 µg/mL. The limit of detection (LOD) and the limit of quantification (LOQ) for the analytical method were determined using FDA guidance on ICH Q2(R1) “*Validation of Analytical Procedures: Text and Methodology: Guidance for Industry*.” Calibration curves for the four chemicals were constructed using seven concentrations levels (0, 1, 2.5, 5, 10, 15, and 20 µg/mL) to establish the LOD and LOQ of the GC-MS analytical method [22].

During GC-MS and LC-MS analyses, a blank solution of each chemical subset was injected between different subsets to minimize and monitor potential carryover. The GC-MS signal intensity of each analyte was determined by integrating the peak area under the total ion chromatogram (TIC). TICs of each analyte were collected, and the background was subtracted using blank samples before calculating the integrated peak area under TIC. The RRF for each analyte was determined by calculating the ratio of its normalized peak area to the peak area of the internal standard.

### LC-MS analysis

Liquid chromatography-mass spectrometry was performed using Thermo Vanquish LC system hybridized with a Thermo Q Exactive HF mass spectrometer equipped with a heated electrospray ionization (HESI) source (ThermoElectron, Waltham, MA, USA). Vanquish LC system was comprised of an autosampler, a binary pump, a UV detector, and a thermostatted column compartment. Separation was performed on Zorbax Eclipse Plus C18 column (2.1 × 100 mm, 1.8 µm), protected by Zorbax Eclipse Plus C18 guard column (2.1 × 5 mm, 1.8 µm) purchased from Agilent. Dry nitrogen was used to create and optimize the spray and ultrapure N_2_ (99.999%) was used as a collision gas when fragmentation was needed. For positive ion detection mode, water with 0.1% formic acid (mobile phase A) and acetonitrile with 0.1% formic acid (mobile phase B) were used. Both the solvent composition and flow rate were programmed as gradient. The solvent gradient increased mobile phase B from 2% after an initial isocratic period of 5 min to reach 35% at 20 min. Then mobile phase B was increased from 35% at 20 min to 100% at 40 min and held on isocratic at 100% from 40 min to 50 min. Then 100% at 50 min was decreased to 2% at 55 min and held for 5 min to equilibrate the column again. The flow rate was kept at 0.4 mL/min through analysis. Specifically, for samples with very low logP (e.g., RM64 and RM96) that have weak interaction with non-polar stationary phases in the reversed-phase column, a considerably slower gradient program was employed to increase their retention on the column. The mobile phase gradient began at 0% B for 5 min, then increased linearly to 10% B by 15 min, and finally reached 100% B at 25 min. The column was held at 100% B for 5 min before decreasing linearly to 2% B by 35 min. After an additional 5 min of equilibration at 2% B, the initial mobile phase composition was restored for 5 min before the next sample injection. The HESI spray voltage in positive ion detection mode was set to 3.8 kV, and the capillary temperature was set to 320 °C for all experiments. During LC-MS analysis, the precolumn and column heater temperature were set to 40 °C. In negative ion mode detection, the same mobile phase composition and gradient program were utilized to test feasibility of formic acid in negative ion mode. The HESI spray voltage of negative ion detection mode was −3.5 kV, and the capillary temperature was set to 340 °C. Like GC-MS analysis, blank solutions were injected both before and after sample injection to avoid and monitor the sample carryover. For each analysis, 2 µL of sample was programmed to be injected onto the column. The sample injection needle was rinsed pre- and post-injection with methanol. LC-MS data acquisition was performed in full scan mode, using both positive and negative ionization modes separately. The mass spectrometry resolution was set to 45,000, with the AGC target for full scan MS set to 1.00E + 06. The m/z scan range was set from 100 to 1500 during the entire study. No in-source CID fragmentation or MS/MS analysis was performed. Each sample was analyzed in triplicate on three different days. Raw data were analyzed using Thermo FreeStyle^TM^ 1.8 software.

For the performance evaluation of LC-MS analysis, three representative reference standards (RM81 and RM89 for positive, and RM 92 for negative ion mode) were employed to monitor the within-day repeatability and the between-days reproducibility. The samples were measured in triplicate at three concentration levels (5, 10, and 20 µg/mL) over three different days. The LOD and LOQ for the LC-MS method were determined from the calibration curves of four representative chemicals (RM8, RM81, and RM89 for positive, and RM92 for negative ion mode) at seven concentration levels (0, 1, 2.5, 5, 10, 15, and 20 µg/mL) over three different days.

GraphPad Prism Version 10.2.3 and embedded statistical functions of Microsoft Excel 365 were employed to calculate the Pearson correlation coefficients of the data obtained from GC-MS and LC-MS analysis.

LC-MS data analysis utilized EIC generated from multiple fragment ions of the parent molecule, as identified in full scan mode. The fragment ions selected for EIC were mainly based on publicly available reference spectra, primarily PubChem, mzCloud^TM^, and other published literatures. To streamline and confirm the quality of EIC, the range of signal intensities of selected ions is between 10 and 100% of the base peak intensity within a defined *m/z* window and their signal-to-noise (S/N) ratio are at least 3 or above.

### Toxicological hazard ranking

A toxicological risk assessment of the medical device relies on the identity and quantity of E&L substituents. Therefore, accounting for the toxicological hazard of the reference standard when selecting the surrogate standards can help generate more realistic E&L profiles in medical devices. Individual hazard endpoints relevant to medical devices were ranked according to severity (Unknown, Low, Moderate, High, or Very High) as indicated in Table S7 using databases shown in Table S8. Rankings were adapted from criteria from the commonly used green chemistry benchmarking tool established in Clean Product Action’s GreenScreen® for Safer Chemicals v1.4 [21] which provides cutoffs for hazard rankings based on Globally Harmonized System (GHS) hazard classification or other available toxicological data [20]. The chemical hazard assessment framework used here incorporated additional endpoints (hemocompatibility and material-medicated pyrogenicity) and excluded environmental, neurotoxicity, and endocrine disruption endpoints included in GreenScreen® for Safer Chemicals [21]. For medical devices, neurotoxicity and endocrine disruption are included in systemic toxicity evaluations; thus, we did not rank those endpoints separately. In addition, all routes of exposure (e.g., intravenous, oral, dermal, inhalation) were considered, and the highest severity (i.e., most toxic) was used for the overall hazard ranking since all are relevant for medical devices. For developmental toxicity, reproductive toxicity, carcinogenicity, and mutagenicity, we added in silico prediction. This was especially important for mutagenicity, a highly weighted endpoint, to reduce the number of substances for which a hazard ranking could not be determined. For substances lacking data on genotoxicity, mutagenicity, and carcinogenicity, predictions were made following ISO/TS 21726 guidelines using OECD QSAR Toolbox v4.6, and VEGA predictions [23–25]. We evaluated material-mediated pyrogenicity based on its presence on the list of pyrogens in ISO 10993-11, Annex G [26]. For hemocompatibility we considered principles in ISO 10993-4 (including hemolysis, complement activation, and thrombogenicity) [27]. Where possible, criteria laid out in GreenScreen^®^ for Safer Chemicals v1.4 was used. Any modifications to this approach are presented in Table S7.

The overall hazard ranking assigned hazards with a higher potential for harm (e.g., carcinogenicity) as Group I with a greater weight. Less severe hazards (e.g., systemic toxicity, sensitization) were classified as Group II carried a medium weight. Acute toxicity and irritation (Group III) had the lowest weight. Individual hazard rankings were combined into an overall hazard ranking, including 1 (high hazard), 2 (moderate hazard), 3 (low hazard with uncertainty), 4 (low hazard), or U (unknown due to insufficient data) as shown in Table S10. Details for endpoint grouping and overall ranking methods are shown in Tables S9 and S10.

## Results

A reference standard set consisting of 106 polymer additives was established for profiling E&Ls in polymeric medical device materials. The included physicochemical properties of selected chemicals were double bond equivalence (DBE), molecular weight (MW), boiling points (BP), acid dissociation constant (pKa), partition coefficient (logP), and refractive index (RI). The physicochemical properties examined in this study span the following ranges: DBE from −2.0 to 25.0, MW from 102.1 to 1177.7 g/mol, BP from 148.3 to 922.0 °C (at 760 mmHg), pKa from −9.1 to 18.25, logP from −0.7 to 23.0, and RI from 1.289 to 1.757. The distributions of three important physicochemical properties — MW, BP, and logP — for 106 reference standards are presented in Fig. S2. In terms of MW, more than 50% of chemicals fell between 200 and 400 g/mol. In this study, most of BP ranged from 300 to 500 °C. BP is a key factor affecting signal response in GC-MS analysis. The proposed reference standards include a broad range of volatile and semi-volatile organic compounds, making them well-suited for our GC-MS analysis. Similarly, logP values, which are one of the critical components for chromatographic separation in the reversed phase column, fell between 2 and 10. Following the reference standard selection criteria defined in the introduction, the reference standards were chosen to represent a wide range of physicochemical properties. To ensure comprehensive coverage in the analysis of all samples, each reference standard was assigned to either GC-MS and/or LC-MS based on predicted applicability derived from the structural information, as well as a priori knowledge of reference mass spectra obtained from PubChem, the NIST database, mzCloud^TM^, Scifinder^n^, and other peer-reviewed literature.

To evaluate the precision of our analytical method and ensure consistent data quality, we assessed both repeatability and reproducibility. Repeatability (or within-day variation) was determined by analyzing triplicates of each concentration (5, 10, and 20 µg/mL) within a single day. A reproducibility (or between-day variation) test was performed over three different days using separately prepared samples. The performance assessment of the analytical method is expressed as within-day and between-day variations in Tables S2A, B.

In GC-MS analysis, the within-day variation (repeatability) was very tight, and the overall between-day variations (reproducibility) of the method were less than 13% (Table S2A). The within-day and between-day variations in LC-MS analysis were also tight. The overall reproducibility measured in positive ion detection was less than 16% and while it was less than 11% in negative mode (Table S2B).

The limit of detection (LOD) and quantification (LOQ) in GC-MS and LC-MS were calculated using the slope and standard deviation of response from the calibration curves of the representative reference standards according to FDA guidance “Q2(R1) Validation of Analytical Procedures: Text and Methodology Guidance for Industry” [22]. Briefly, the equations used to calculate LOD and LOQ in this study are expressed as:

$${LOD}=3.3{{{\rm{\sigma }}}}/{{{\rm{m}}}}$$

$${LOQ}=10\sigma /{{{\rm{m}}}}$$

Where σ is the standard deviation of the response (i.e., y-intercept from linear regression) and m is the slope of the calibration curve. The calculated LOD and LOQ values for GC-MS and LC-MS analyses including calibration range, linearity, and R^2^ values for each calibration standard are described in Table S3. The calibration curve test was conducted on at least three separate days prior to sample injection.

In GC-MS analysis, the LOD ranged from 0.7 to 2.1 µg/mL, and the LOQ ranged from 2.2 to 6.4 µg/mL for the four selected chemicals (RM5, RM33, RM52 and RM66) that represent the reference standards with weak and medium signal response in GC-MS. While in LC-MS analysis, three chemicals (RM8, RM81, and RM89) for positive ion mode and one chemical (RM92) for negative ion mode were selected to establish the calibration curves and the calculated LOD and LOQ range from 1.3 to 2.3 µg/mL and 4.1 to 7.0 µg/mL, respectively. The RRF values obtained from the GC-MS analysis are presented in Table 1. For the LC-MS analysis, RRF values were determined in both positive and negative ion detection modes and are reported in Tables 2 and 3, respectively. For both analysis methods, the peak area of corresponding chromatograms was employed to calculate the RRF values for quantifiable reference standards. However, for chemicals exhibiting weak chromatographic responses, the corresponding RRF values are indicated as either *‘below detection limit (below DL)*’ or *‘not detected (n.d.)’* in Tables 1, 2, and 3. A value is categorized as *below DL*, when the S/N ratio in the chromatogram is below 3, but the discernable peak is still present. In contrast, a value marked as *n.d*. when the S/N ratio is less than 1 and the peak cannot be distinguished from the background noise.

**Table 1:** RRF values for 92 reference standards were determined using GC-MS.

Code	IUPAC Name	CAS #	RRF values from TIC of GC-MS (Positive Ion Detection Mode)		
			5 µg/mL	10 µg/mL	20 µg/mL
**RM1**	[3-[3-(3,5-ditert-butyl-4-hydroxyphenyl)propanoyloxy]-2,2-bis[3-(3,5-ditert-butyl-4-hydroxyphenyl)propanoyloxymethyl]propyl] 3-(3,5-ditert-butyl-4-hydroxyphenyl)propanoate	6683-19-8	*n.d*.	*n.d*.	*n.d*.
**RM2**	octadecyl 3-(3,5-ditert-butyl-4-hydroxyphenyl)propanoate	2082-79-3	0.326	0.434	0.336
**RM3**	tris(2,4-ditert-butylphenyl) phosphite	31570-04-4	0.414	0.393	0.281
**RM4**	2,6-ditert-butyl-4-methylphenol	128-37-0	1.828	2.143	1.81
**RM5**	2,4-ditert-butylphenol	96-76-4	**1**	**1**	**1**
**RM6**	2,4-dimethylphenol	105-67-9	0.71	0.377	0.424
**RM7**	2-tert-butyl-4-methoxyphenol	25013-16-5	0.606	0.506	0.583
**RM8**	bis(2-ethylhexyl) benzene-1,2-dicarboxylate	117-81-7	0.823	0.824	0.838
**RM9**	dibutyl benzene-1,2-dicarboxylate	84-74-2	0.713	0.744	0.822
**RM10**	diphenyl benzene-1,2-dicarboxylate	84-62-8	0.18	0.437	0.676
**RM11**	2-O-benzyl 1-O-butyl benzene-1,2-dicarboxylate	85-68-7	0.364	0.383	0.476
**RM12**	tris(2-ethylhexyl) benzene-1,2,4-tricarboxylate	3319-31-1	0.503	0.47	0.525
**RM13**	azepan-2-one	105-60-2	0.214	0.289	0.413
**RM14**	4-[2-(4-hydroxyphenyl)propan-2-yl]phenol	80-05-7	0.066	0.241	0.314
**RM15**	(Z)-docos-13-enamide	112-84-5	0.143	0.165	0.086
**RM16**	(Z)-octadec-9-enamide	301-02-0	0.033	0.035	0.04
**RM17**	butyl octadecanoate	123-95-5	0.295	0.288	0.275
**RM18**	2-(benzotriazol-2-yl)-4,6-ditert-butylphenol	3846-71-7	0.182	0.575	0.723
**RM19**	2,2,4,4,6,6,8,8-octamethyl-1,3,5,7,2,4,6,8-tetraoxatetrasilocane	556-67-2	1.262	1.828	1.537
**RM20**	2,2,4,4,6,6,8,8,10,10,12,12-dodecamethyl-1,3,5,7,9,11-hexaoxa-2,4,6,8,10,12-hexasilacyclododecane	540-97-6	1.076	0.773	1.134
**RM21**	bis(2-methylpropyl) hexanedioate	141-04-8	0.663	0.609	0.625
**RM22**	bis(6-methylheptyl) nonanedioate	26544-17-2	*n.d*.	*n.d*.	*n.d*.
**RM23**	dibutyl decanedioate	109-43-3	0.377	0.393	0.451
**RM24**	1-O-heptyl 6-O-nonyl hexanedioate	68515-75-3	0.014	0.022	0.023
**RM25**	N-benzyl-1-phenylmethanamine	103-49-1	0.654	0.741	0.896
**RM26**	benzoic acid	65-85-0	*below DL*	*below DL*	0.035
**RM27**	2-ethylhexanoic acid	149-57-5	0.084	0.121	0.166
**RM28**	1-chloro-4-(4-chlorophenyl)sulfonylbenzene	80-07-9	0.707	0.733	0.725
**RM29**		88-26-6	0.297	0.282	0.31
	2,6-ditert-butyl-4-(hydroxymethyl)phenol				
**RM30**	1,3-ditert-butylbenzene	1014-60-4	2.551	2.006	1.653
**RM31**	tris(2,4-ditert-butylphenyl) phosphate	95906-11-9	0.206	0.172	0.175
**RM32**	hexadecanoic acid	57-10-3	*n.d*.	*n.d*.	*n.d*.
**RM33**	N-(1,3-benzothiazol-2-ylsulfanyl)-2-methylpropan-2-amine	95-31-8	0.457	0.442	0.58
**RM34**	1,2-bis(2-methylphenyl)guanidine	97-39-2	*below DL*	*below DL*	*below DL*
**RM35**	1-N-phenyl-4-N-propan-2-ylbenzene-1,4-diamine	101-72-4	0.176	0.201	0.214
**RM37**	5-chloro-2-(2,4-dichlorophenoxy)phenol	3380-34-5	0.101	0.136	0.15
**RM40**	2,5,7,8-tetramethyl-2-(4,8,12-trimethyltridecyl)-3,4-dihydrochromen-6-ol	10191-41-0	0.121	0.144	0.142
**RM41**	2-tert-butyl-6-[(3-tert-butyl-5-ethyl-2-hydroxyphenyl)methyl]-4-ethylphenol	88-24-4	0.208	0.297	0.378
**RM44**	2-[2-[3-(3,5-ditert-butyl-4-hydroxyphenyl)propanoyloxy]ethylsulfanyl]ethyl 3-(3,5-ditert-butyl-4-hydroxyphenyl)propanoate	41484-35-9	0.099	0.152	0.022
**RM47**	N,N-bis(2-hydroxyethyl)dodecanamide	120-40-1	*n.d*.	*n.d*.	*n.d*.
**RM48**	2,3-dihydroxypropyl octadecanoate	123-94-4	*n.d*.	*n.d*.	*n.d*.
**RM49**	1,3-benzothiazole	95-16-9	0.863	0.891	0.857
**RM50**	N,N-dimethyl-1-phenylmethanamine	103-83-3	1.53	1.467	1.358
**RM51**	4-tert-butylphenol	98-54-4	0.687	0.724	0.737
**RM52**	4-(2-methylbutan-2-yl)phenol	80-46-6	0.615	0.527	0.606
**RM53**	4-[(4-aminophenyl)methyl]aniline	101-77-9	0.142	0.243	0.377
**RM55**	N-(1,3-benzothiazol-2-ylsulfanyl)cyclohexanamine	95-33-0	0.104	0.107	0.149
**RM56**	3H-1,3-benzothiazole-2-thione	149-30-4	*below DL*	*below DL*	*below DL*
**RM57**	octadecanoic acid	57-11-4	*below DL*	*below DL*	*below DL*
**RM58**	1,4-diphenylbenzene	92-94-4	1.508	1.98	1.768
**RM59**	N-[2-(octadecanoylamino)ethyl]octadecanamide	110-30-5	*n.d*.	*n.d*.	*n.d*.
**RM60**	3,4-dimethylbenzaldehyde	5973-71-7	0.734	0.866	0.967
**RM61**	2,2-dimethyl-N-phenylpropanamide	6625-74-7	0.632	0.661	0.69
**RM63**	triethyl phosphate	78-40-0	0.274	0.297	0.355
**RM64**	2,3-diacetyloxypropyl acetate	102-76-1	0.302	0.337	0.383
**RM65**	triethyl 2-hydroxypropane-1,2,3-tricarboxylate	77-93-0	0.106	0.145	0.205
**RM66**	[2,2,4-trimethyl-3-(2-methylpropanoyloxy)pentyl] 2-methylpropanoate	6846-50-0	0.708	0.674	0.668
**RM67**	methyl (Z)-octadec-9-enoate	112-62-9	0.377	0.378	0.396
**RM68**	propan-2-yl hexadecanoate	142-91-6	0.468	0.451	0.616
**RM69**	diphenyl benzene-1,4-dicarboxylate	1539-04-4	0.14	0.259	0.245
**RM70**	6-amino-4-(ethylamino)-1H-1,3,5-triazin-2-one	7313-54-4	*n.d*.	*n.d*.	*n.d*.
**RM71**	tributyl 2-hydroxypropane-1,2,3-tricarboxylate	77-94-1	0.022	0.099	0.163
**RM72**	bis(6-methylheptyl) benzene-1,2-dicarboxylate	27554-26-3	*below DL*	*below DL*	*below DL*
**RM73**	bis(2-ethylhexyl) benzene-1,4-dicarboxylate	6422-86-2	0.266	0.292	0.265
**RM74**	tributyl 2-acetyloxypropane-1,2,3-tricarboxylate	77-90-7	0.333	0.356	0.347
**RM75**	dioctyl nonanedioate	2064-80-4	0.042	0.038	0.037
**RM76**	bis(2-ethylhexyl) decanedioate	122-62-3	0.175	0.286	0.238
**RM77**	bis(7-methyloctyl) benzene-1,2-dicarboxylate	28553-12-0	*below DL*	*below DL*	*below DL*
**RM79**	bis(8-methylnonyl) hexanedioate	27178-16-1	*n.d*.	*n.d*.	*n.d*.
**RM80**	dicyclohexyl benzene-1,2-dicarboxylate	84-61-7	0.51	0.527	0.443
**RM83**	bis(2-ethylhexyl) cyclohexane-1,2-dicarboxylate	84-71-9	0.277	0.294	0.274
**RM85**	2-benzofuran-1,3-dione	85-44-9	*below DL*	*below DL*	*below DL*
**RM86**	2H-benzotriazole	95-14-7	*below DL*	*below DL*	*below DL*
**RM87**	2-(1,3-benzothiazol-2-yl)phenol	3411-95-8	0.09	0.14	0.163
**RM88**	2-tert-butyl-6-(5-chlorobenzotriazol-2-yl)-4-methylphenol	11/5/3896	0.135	0.142	0.154
**RM89**	(2-hydroxy-4-octoxyphenyl)-phenylmethanone	1843-05-6	0.161	0.233	0.22
**RM90**	2-(benzotriazol-2-yl)-4,6-bis(2-methylbutan-2-yl)phenol	25973-55-1	0.401	0.394	0.285
**RM91**	bis(2,2,6,6-tetramethylpiperidin-4-yl) decanedioate	52829-07-9	0.296	0.312	0.317
**RM92**	(2,4-dihydroxyphenyl)-phenylmethanone	131-56-6	*below DL*	*below DL*	0.035
**RM93**	2-(benzotriazol-2-yl)-4-methylphenol	2440-22-4	0.189	0.22	0.23
**RM94**	2-(benzotriazol-2-yl)-4-(2,4,4-trimethylpentan-2-yl)phenol	3147-75-9	0.196	0.21	0.166
**RM95**	2-(benzotriazol-2-yl)-4,6-bis(2-phenylpropan-2-yl)phenol	70321-86-7	0.255	0.253	0.172
**RM96**	imidazolidine-2-thione	96-45-7	*below DL*	0.031	0.058
**RM97**	2,2,3,3,4,4,5,5,6,6,7,7,8,8,9,9,10,10,10-nonadecafluorodecanoic acid	335-76-2	*n.d*.	*n.d*.	*n.d*.
**RM98**	2-ethylhexan-1-ol	104-76-7	0.472	0.547	0.551
**RM99**	azacyclotridecan-2-one	947-04-6	0.17	0.219	0.25
**RM100**	tetradecane	629-59-4	1.339	1.572	1.535
**RM101**	2-hydroxy-2-methyl-1-phenylpropan-1-one	7473-98-5	0.274	0.347	0.4
**RM102**	2-phenylpropan-2-ol	617-94-7	0.728	0.737	0.691
**RM103**	dodec-1-ene	112-41-4	1.444	1.765	1.645
**RM105**	dodecyl 3-(3-dodecoxy-3-oxopropyl)sulfanylpropanoate	123-28-4	*n.d*.	*n.d*.	*n.d*.
**RM106**	2-(2-ethoxyethoxy)ethanol	111-90-0	*below DL*	0.028	0.088

The internal standard is highlighted in bold. (*n.d*.: *not detected*, *below DL*: *below the detection limit*, where the peak may be discernible but the signal-to-noise ratio of the sample peak in the TIC was less than 3).

**Table 2:** RRF values for 57 reference standards were determined using LC-MS in positive ion detection mode.

Code	IUPAC Name	CAS #	RRF values from EIC of LC-MS (Positive Ion Detection Mode)		
			5 µg/mL	10 µg/mL	20 µg/mL
**RM1**	[3-[3-(3,5-di*tert*-butyl-4-hydroxyphenyl)propanoyloxy]-2,2-bis[3-(3,5-di*tert*-butyl-4-hydroxyphenyl)propanoyloxymethyl]propyl] 3-(3,5-di*tert*-butyl-4-hydroxyphenyl)propanoate	6683-19-8	0.557	0.537	0.521
**RM2**	octadecyl 3-(3,5-di*tert*-butyl-4-hydroxyphenyl)propanoate	2082-79-3	0.012	0.019	0.017
**RM4**	2,6-di*tert*-butyl-4-methylphenol	128-37-0	0.32	0.39	0.435
**RM5**	2,4-di*tert*-butylphenol	96-76-4	*n.d*.	*n.d*.	*n.d*.
**RM8**	bis(2-ethylhexyl) benzene-1,2-dicarboxylate	117-81-7	0.65	0.552	0.635
**RM13**	azepan-2-one	105-60-2	12.128	10.499	13.718
**RM14**	4-[2-(4-hydroxyphenyl)propan-2-yl]phenol	80-05-7	*n.d*.	*n.d*.	*n.d*.
**RM15**	(*Z*)-docos-13-enamide	112-84-5	3.895	4.691	5.387
**RM16**	(*Z*)-octadec-9-enamide	301-02-0	8.755	6.478	6.468
**RM17**	butyl octadecanoate	123-95-5	0.003	0.003	0.003
**RM22**	bis(6-methylheptyl) nonanedioate	26544-17-2	1.78	1.84	1.879
**RM25**	*N*-benzyl-1-phenylmethanamine	103-49-1	10.305	9.543	10.708
**RM26**	benzoic acid	65-85-0	*below DL*	0.039	0.055
**RM27**	2-ethylhexanoic acid	149-57-5	0.034	0.04	0.054
**RM28**	1-chloro-4-(4-chlorophenyl)sulfonylbenzene	80-07-9	0.398	0.455	0.571
**RM29**	2,6-di*tert*-butyl-4-(hydroxymethyl)phenol	88-26-6	*n.d*.	*n.d*.	*n.d*.
**RM30**	1,3-di*tert*-butylbenzene	1014-60-4	*n.d*.	*n.d*.	*n.d*.
**RM31**	tris(2,4-di*tert*-butylphenyl) phosphate	95906-11-9	0.779	0.556	0.423
**RM32**	hexadecanoic acid	57-10-3	*n.d*.	*n.d*.	*n.d*.
**RM34**	1,2-bis(2-methylphenyl)guanidine	97-39-2	2.239	2.468	2.778
**RM35**	1-*N*-phenyl-4-*N*-propan-2-ylbenzene-1,4-diamine	101-72-4	1.558	1.817	2.111
**RM36**	1,3,5-tris[(3,5-di*tert*-butyl-4-hydroxyphenyl)methyl]-1,3,5-triazinane-2,4,6-trione	27676-62-6	0.033	0.029	0.024
**RM38**	5-chloro-2-(2,4-dichlorophenoxy)phenol	13463-41-7	*below DL*	*below DL*	*below DL*
**RM39**	zinc;1-oxidopyridin-1-ium-2-thiolate	199111-50-7	*n.d*.	*n.d*.	*n.d*.
**RM40**	dimethyl-octadecyl-(3-trihydroxysilylpropyl)azanium;chloride	10191-41-0	0.149	0.172	0.19
**RM42**	4-(1-phenylethyl)-*N*-[4-(1-phenylethyl)phenyl]aniline	60160-25-0	0.881	1.073	1.408
**RM43**	(piperidine-1-carbothioyltrisulfanyl) piperidine-1-carbodithioate	120-54-7	*n.d*.	*n.d*.	*n.d*.
**RM45**	3,9-bis[2,4-bis(2-phenylpropan-2-yl)phenoxy]-2,4,8,10-tetraoxa-3,9-diphosphaspiro[5.5]undecane	154862-43-8	*n.d*.	*n.d*.	*n.d*.
**RM46**	2-[3,5-bis[2-[3-(3,5-di*tert*-butyl-4-hydroxyphenyl)propanoyloxy]ethyl]-2,4,6-trioxo-1,3,5-triazinan-1-yl]ethyl 3-(3,5-di*tert*-butyl-4-hydroxyphenyl)propanoate	34137-09-2	1.14	0.947	0.846
**RM47**	*N*,*N*-bis(2-hydroxyethyl)dodecanamide	120-40-1	0.661	0.764	0.869
**RM48**	2,3-dihydroxypropyl octadecanoate	123-94-4	0.028	0.031	0.038
**RM49**	1,3-benzothiazole	95-16-9	0.293	0.396	0.533
**RM54**	6-(dibutylamino)-1H-1,3,5-triazine-2,4-dithione	29529-99-5	0.099	0.162	0.247
**RM56**	3*H*-1,3-benzothiazole-2-thione	149-30-4	0.702	0.604	0.734
**RM57**	octadecanoic acid	57-11-4	*n.d*.	*n.d*.	*n.d*.
**RM62**	sodium;1,3,7,9-tetra*tert*-butyl-11-oxido-5*H*-benzo[d][1,3,2]benzodioxaphosphocine 11-oxide	85209-91-2	1.38	1.337	1.531
**RM63**	triethyl phosphate	78-40-0	1.914	2.272	2.599
**RM64**	2,3-diacetyloxypropyl acetate	102-76-1	0.28	0.343	0.4
**RM65**	triethyl 2-hydroxypropane-1,2,3-tricarboxylate	77-93-0	1.36	1.443	1.506
**RM72**	bis(6-methylheptyl) benzene-1,2-dicarboxylate	27554-26-3	0.496	0.481	0.518
**RM74**	tributyl 2-acetyloxypropane-1,2,3-tricarboxylate	77-90-7	0.903	1.014	1.085
**RM77**	bis(7-methyloctyl) benzene-1,2-dicarboxylate	28553-12-0	0.768	0.775	0.754
**RM78**	bis(7-methyloctyl) cyclohexane-1,2-dicarboxylate	166412-78-8	1	1.091	1.129
**RM79**	bis(8-methylnonyl) hexanedioate	27178-16-1	0.992	1.076	1.197
**RM81**	**trihexyl 2-butanoyloxypropane-1,2,3-tricarboxylate**	**82469-79-2**	**1**	**1**	**1**
**RM82**	tris(6-methylheptyl) benzene-1,2,4-tricarboxylate	27251-75-8	*n.d*.	*n.d*.	*n.d*.
**RM84**	Tri(n-octyl, n-decyl) trimellitate	67989-23-5	0.834	0.676	0.736
**RM85**	2-benzofuran-1,3-dione	85-44-9	0.004	0.004	0.005
**RM86**	2*H*-benzotriazole	95-14-7	0.551	0.686	0.884
**RM89**	(2-hydroxy-4-octoxyphenyl)-phenylmethanone	1843-05-6	0.249	0.321	0.458
**RM93**	2-(benzotriazol-2-yl)-4-methylphenol	2440-22-4	0.66	0.713	0.843
**RM94**	2-(benzotriazol-2-yl)-4-(2,4,4-trimethylpentan-2-yl)phenol	3147-75-9	0.238	0.305	0.423
**RM96**	imidazolidine-2-thione	96-45-7	0.091	0.154	0.249
**RM99**	azacyclotridecan-2-one	947-04-6	1.291	1.578	1.871
**RM104**	2-dodecylbenzenesulfonic acid	27176-87-0	*n.d*.	*n.d*.	*n.d*.
**RM105**	dodecyl 3-(3-dodecoxy-3-oxopropyl)sulfanylpropanoate	123-28-4	0.313	0.321	0.327
**RM106**	2-(2-ethoxyethoxy)ethanol	111-90-0	0.489	0.602	0.713

The internal standard is highlighted in bold. (*n.d*.: *not detected*, *below DL*: *below the detection limit*, where the peak may be discernible but the signal-to-noise ratio of the sample peak in the EIC was less than 3).

**Table 3:** RRF values for 28 reference standards were determined using LC-MS in negative ion detection mode.

Code	IUPAC Name	CAS #	RRF from EIC of LC-MS (Negative Ion Detection Mode)		
			5 µg/mL	10 µg/mL	20 µg/mL
**RM2**	octadecyl 3-(3,5-ditert-butyl-4-hydroxyphenyl)propanoate	2082-79-3	*n.d*.	*n.d*.	*n.d*.
**RM4**	2,6-di*tert*-butyl-4-methylphenol	128-37-0	*below DL*	*below DL*	*below DL*
**RM5**	2,4-di*tert*-butylphenol	96-76-4	*below DL*	*below DL*	*below DL*
**RM8**	bis(2-ethylhexyl) benzene-1,2-dicarboxylate	117-81-7	*n.d*.	*n.d*.	*n.d*.
**RM13**	azepan-2-one	105-60-2	*n.d*.	*n.d*.	*n.d*.
**RM14**	4-[2-(4-hydroxyphenyl)propan-2-yl]phenol	80-05-7	*n.d*.	*n.d*.	*n.d*.
**RM15**	(*Z*)-docos-13-enamide	112-84-5	*n.d*.	*n.d*.	*n.d*.
**RM16**	(*Z*)-octadec-9-enamide	301-02-0	*n.d*.	*n.d*.	*n.d*.
**RM25**	*N*-benzyl-1-phenylmethanamine	103-49-1	*n.d*.	*n.d*.	*n.d*.
**RM26**	benzoic acid	65-85-0	*n.d*.	*n.d*.	*n.d*.
**RM27**	2-ethylhexanoic acid	149-57-5	*n.d*.	*n.d*.	*n.d*.
**RM28**	1-chloro-4-(4-chlorophenyl)sulfonylbenzene	80-07-9	*n.d*.	*n.d*.	*n.d*.
**RM29**	2,6-di*tert*-butyl-4-(hydroxymethyl)phenol	88-26-6	0.002	0.003	0.003
**RM30**	1,3-di*tert*-butylbenzene	1014-60-4	*n.d*.	*n.d*.	*n.d*.
**RM31**	tris(2,4-di*tert*-butylphenyl) phosphate	95906-11-9	*n.d*.	*n.d*.	*n.d*.
**RM32**	hexadecanoic acid	57-10-3	*n.d*.	*n.d*.	*n.d*.
**RM37**	5-chloro-2-(2,4-dichlorophenoxy)phenol	3380-34-5	0.314	0.323	0.389
**RM46**	2-[3,5-bis[2-[3-(3,5-ditert-butyl-4-hydroxyphenyl)propanoyloxy]ethyl]-2,4,6-trioxo-1,3,5-triazinan-1-yl]ethyl 3-(3,5-ditert-butyl-4-hydroxyphenyl)propanoate	34137-09-2	0.02	0.017	0.016
**RM56**	3*H*-1,3-benzothiazole-2-thione	149-30-4	0.384	0.448	0.448
**RM57**	octadecanoic acid	57-11-4	*n.d*.	*n.d*.	*n.d*.
**RM62**	sodium;1,3,7,9-tetratert-butyl-11-oxido-5H-benzo[d][1,2,3]benzodioxaphosphocine 11-oxide	85209-91-2	0.869	0.951	0.997
**RM70**	6-amino-4-(ethylamino)-1H-1,3,5-triazin-2-one	7313-54-4	0.005	0.007	0.01
**RM82**	tris(6-methylheptyl) benzene-1,2,4-tricarboxylate	27251-75-8	*n.d*.	*n.d*.	*n.d*.
**RM84**	Tri(n-octyl, n-decyl) trimellitate	67989-23-5	*n.d*.	*n.d*.	*n.d*.
**RM92**	**(2,4-dihydroxyphenyl)-phenylmethanone**	**131-56-6**	**1**	**1**	**1**
**RM93**	2-(benzotriazol-2-yl)-4-methylphenol	2440-22-4	*n.d*.	*n.d*.	*n.d*.
**RM97**	2,2,3,3,4,4,5,5,6,6,7,7,8,8,9,9,10,10,10-nonadecafluorodecanoic acid	335-76-2	0.979	1.072	1.143
**RM104**	2-dodecylbenzenesulfonic acid	27176-87-0	0.246	0.261	0.268

The internal standard is highlighted in bold. (*n.d*.: *not detected*, *below DL*: *below the detection limit*, where the peak may be discernible but the signal-to-noise ratio of the sample peak in the EIC was less than 3).

In GC-MS analysis, the RRF values of 92 reference standards were measured at three different concentrations. The RRF was calculated by comparing the analyte signal to that of the internal standard, RM5 (2,4-Di-t-butyl phenol). Since the samples were analyzed in multiple subsets with the internal standard, the signal response obtained from each subset was normalized to the intensity of internal standard RM5. The overall RRF values for target analytes measured in GC-MS analysis range from 0.014 to 2.551 at three concentrations (5, 10, and 20 µg/mL). For most analytes the RRF values remained relatively consistent across this concentration range.

**Fig. 1:**
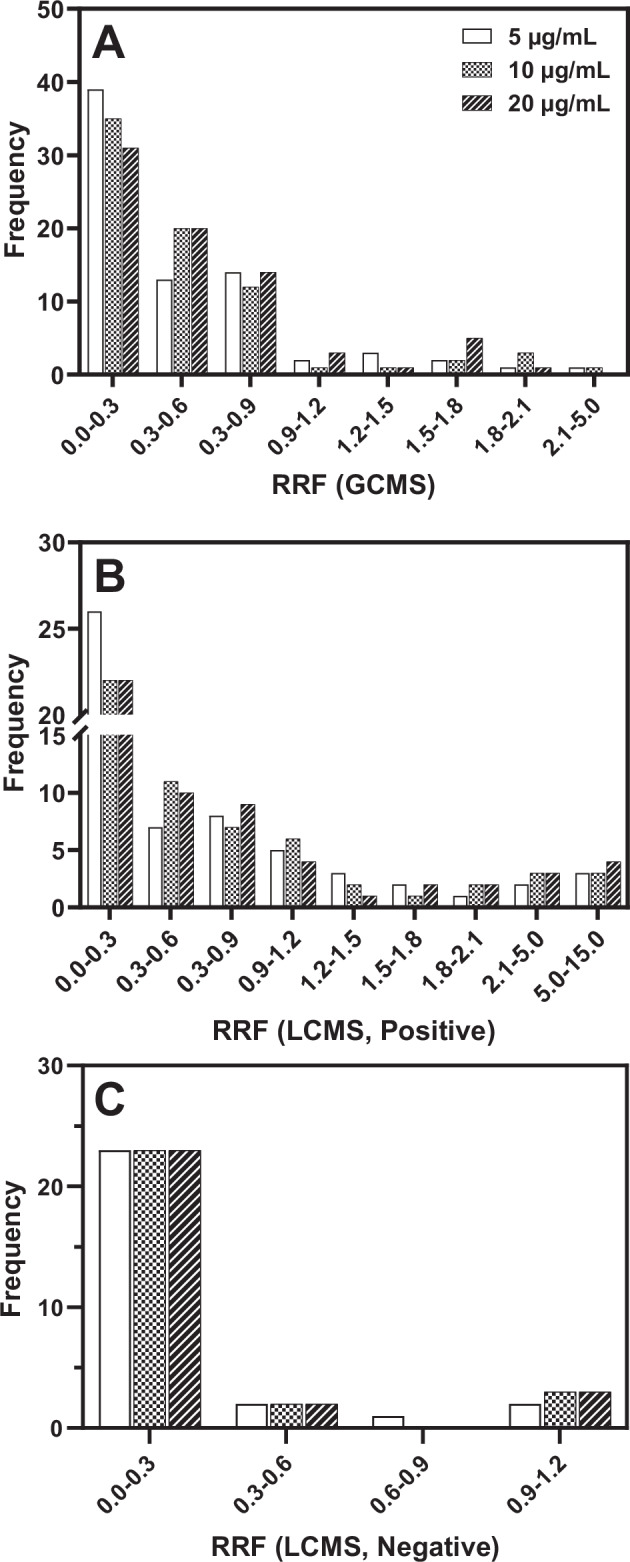
Distribution of Relative Response Factors (RRF) for 106 Reference Standards Across Analytical Platforms. The distribution of relative response factors (RRF) measured in GC-MS analysis **A** and LC-ESI-MS analysis in positive **B** and negative **C** ion detection modes. The relative response factors (RRF) values of all chemicals were measured at three concentrations: 5, 10, and 20 µg/mL.

The distribution of RRF values obtained from GC-MS analysis is shown in Fig. 1A. Seventeen of 92 tested reference standards were denoted either ‘*below DL*’ or ‘*n.d*.’ across three concentrations in GC-MS analyses. Over 70% of RRF values fall within 0 and 1 and the rest of them are between 1 and 3 (Fig. S5A).

For LC-MS analysis, 57 chemicals were tested in positive mode using RM81 as an internal standard, while 28 chemicals were tested in negative mode using RM92 as an internal standard. For the quantitative calculation of RRF, we constructed the EIC using multiple ions that exhibited at least 10% of the base peak signal intensity, along with a minimum S/N ratio of 3. Under these conditions, we found that the RRF values calculated from both TIC and EIC were close each other. Additionally, the use of EIC significantly improved the S/N ratio of the sample peaks in the chromatogram. Like in GC-MS, samples were analyzed in different subsets and the normalized intensities were used to calculate the RRF values. The number of chemicals denoted as ‘*below DL*’ or ‘*n.d*.’ in positive and negative LC-MS analyses are 12 and 19, respectively. The overall RRF values range from 0.003 to 13.718 in positive ion detection mode (Fig. S5B) and 0.002 to 1.143 in negative mode (Fig. S5C) of LC-MS analyses across three different concentrations (5, 10, and 20 µg/mL). The RRF values for each analyte remain relatively constant throughout the concentration range.

The distributions of RRF values measured by positive or negative ion detection mode in LC-MS analysis are shown in Fig. 1B, C, respectively. Almost 70% of RRF values fell between 0 and 1; and the rest of them ranged from 1 to 15. While in negative ion mode, 19 out of 27 reference standards were ‘*below DL*’ or ‘*n.d*.’. We understand that the low detectability of chemicals in negative mode is due to the use of 0.1% formic acid as an additive in the mobile phase. However, in this study, we tested the sensitivity of chemicals with low logP using formic acid to evaluate whether the experimental process could be streamlined. In upcoming study, we plan to retest these chemicals in negative ion mode with different basic additives. All reported RRF values in negative mode were below 1. Overall, in both GC-MS and LC-MS, most RRF values of reference standards show narrow variation at three concentrations.

To assess the relationships between the physicochemical properties and the signal response of individual RMs, we conducted regression and correlation analyses between RRF values obtained from GC-MS and LC-MS data across three concentrations alongside the four physicochemical properties relevant to the detectability of chemicals in GC-MS and LC-MS, including molecular weight, boiling point, logP, and pKa values. The methodology employed is based on published methods [28] using Microsoft Excel Data Analysis Tool and GraphPad Prism (Version 10.2.3). The Pearson correlation coefficient (r) was calculated for each dataset to assess the strength of linear relationship between selected parameters and RRF values. The r value ranges from −1 to +1 where a value near +1 indicates a strong positive linear correlation, and a value near −1 indicates a strong negative correlation.

Table S12 shows the *r* values for the GC-MS data across the three concentrations are less than −0.01 (*p* > 0.05), indicating that the correlation between RRF and either logP or pKa is insignificant. Similar results were observed from the LC-MS data, except for pKa in the positive ion mode. Notably, an *r* value below 0.4 suggests a weak correlation [29]. In the GC-MS data, the *r* values for molecular weight and boiling point ranged from −0.2 to −0.5 across all three concentrations, suggesting a moderate to weak negative correlation between RRF and both molecular weight and boiling point. Additionally, Fig. S8 presents a representative linear regression analysis of the four physicochemical properties and RRF obtained from GC-MS at a concentration of 5 µg/mL. Similarly, the LC-MS data (in both positive and negative ion modes) demonstrated r values between RRF and molecular weight or boiling point ranging from −0.03 to −0.22, indicating very weak correlations with *p*-values exceeding 0.05. Therefore, the overall statistical analysis indicates that there is no significant correlation between the RRF values and the selected four physicochemical properties. The absence of correlation is likely attributable to the complex nature of ionization efficiency and signal response in mass spectrometry [30–36].

**Fig. 2:**
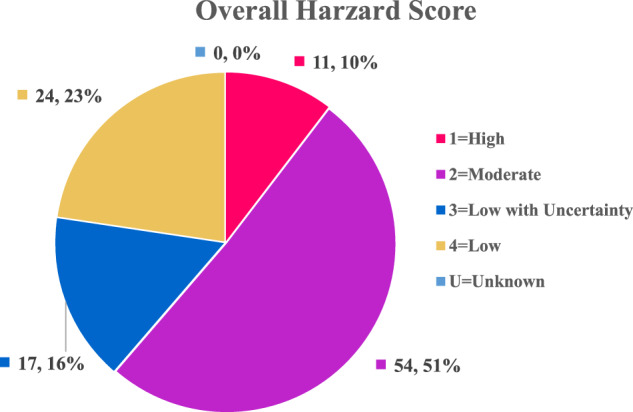
Summary of Overall Hazard Rankings Assigned to 106 Reference Standards. Overall hazard rankings are categorized into five levels: High (1), Moderate (2), Low with Uncertainty (3), Low (4), and Unknown (U). The numbers represent the count out of 106 chemicals and percentage of occurrence in each category. Most classified hazard ranking is moderate and only 10% of chemicals was identified as high hazard. This figure illustrates how the reference standards proposed in this study cover a broad spectrum of toxicological concerns while minimizing the inclusion of highly toxic chemicals.

**Fig. 3:**
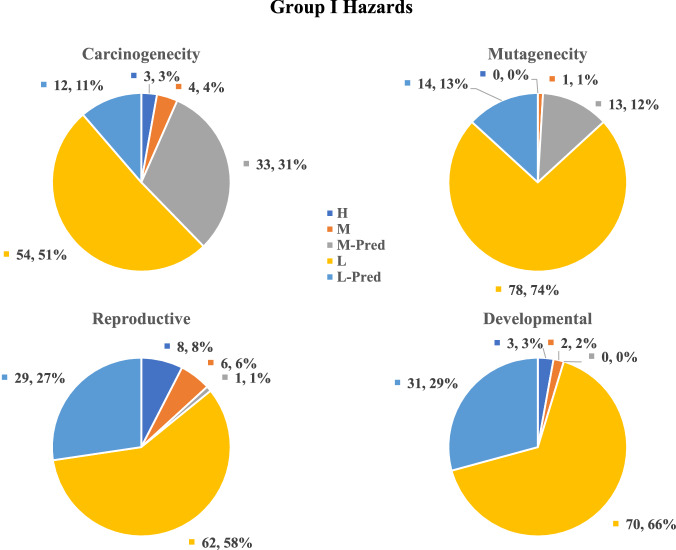
Distribution of Group I Hazard Categories Across 106 Reference Standards. This figure presents the distribution of Group I hazard categories: carcinogenicity, mutagenicity, reproductive, and developmental toxicity. Each category is divided into different levels: high (H), moderate (M), moderate-predicted (M-pred), low (L), low-predicted (L-pred), and Unknown (U). The numbers represent the count and percentage of 106 chemicals at each ranking level within their respective categories.

From a toxicological perspective, the selected RM compounds broadly cover the toxicological endpoints typically assessed in biocompatibility evaluation. Figure 2 shows that the overall hazard ranking categorizes 51% of substances as a moderate hazard (i.e., overall hazard ranking of 2) and 10% as a high hazard (i.e., overall hazard ranking of 1). Figure 3 provides an overview of the evaluated substances’ highly weighted hazard profiles (Group I, carcinogenicity, mutagenicity, reproductive toxicity, and developmental toxicity) that heavily drive overall hazard rankings. Hazards with lower weight (i.e., Group II and III) are shown in Figs. S6 and S7. Only respiratory sensitization and hemocompatibility had a significant percentage ( >20%) of unknown hazard severity (i.e., there was insufficient toxicological data to predict the hazard, or the endpoint could not be predicted using in silico methods). Hazard rankings for individual endpoints exhibited considerable variability, with a tendency toward low hazard rankings — there were more chemicals ranked as low or moderate hazard than high hazard. For individual endpoints, high or very high rankings were not present for mutagenicity, systemic toxicity (acute and repeat), and sensitization (skin and respiratory). Skin sensitization, respiratory sensitization, hemocompatibility, and skin irritation lacked substances with moderate rankings. Table S11 shows chemical hazard rankings for all substances.

## Discussion

Establishing a comprehensive set of reference standards for E&L profiling in medical devices is important for accurate chemical characterization. A standardized approach for correct E&L profiling ensures the reproducibility and accuracy of chemical analysis for E&L constituents in device extracts, which is important for toxicological risk assessments used to evaluate the safety of medical devices as an alternative to in vivo testing. In this study, we proposed appropriately selected reference standards for the semi-quantitation analysis of polymer additives and measured signal responses of those RMs at three different concentrations. Our RRF values in GC-MS and LC-MS analyses demonstrated reproducibility across all three concentrations.

Furthermore, the proposed chemical reference standards encompass a wide range of physicochemical properties and chemical classes, while also providing broad toxicological coverage. Given the critical link between chemical characterization and toxicological risk assessment of medical devices, it is important to ensure that all toxicological endpoints are included in the reference set — intended to be representative of device extractables — to try and minimize any risk that they may be overlooked during the NTA process. Overlooked substances will not undergo toxicological risk assessment and could pose a safety risk to the patient. The presence of few high or very high hazard substances suggests a general bias towards selecting biocompatible materials in medical device design. Additionally, the infrequency of high hazard substances is advantageous, as it reduces the overall risk these compounds pose throughout a device’s life cycle — from the manufacturing process to the chemists conducting E&L studies, and ultimately to the patient and user. The absence of high or very high hazard substances for sensitization, material-mediated pyrogenicity, and respiratory sensitization does not negatively affect the utility of the set of RM because these endpoints are typically evaluated through other means (e.g., biological testing). For material-mediated pyrogenicity, this finding is consistent with the rare occurrence of material-mediated pyrogenicity. Future efforts will evaluate whether highly toxic substances, so-called cohort of concern (CoC) substances, identified in ISO/TS 21726 are adequately covered by the reference standard list [23]. These substances are explicitly excluded from NTA per ISO 10993-18; however, understanding CoC coverage may help define the limitations of this list of reference standards and provide additional assurance that these highly toxic substances can be identified if they are present above the AET [5, 23, 37].

To measure the sample intensities, we employed TIC for GC-MS and EIC for LC-MS to minimize interference from the background noise and enhance S/N ratio. In GC-MS, high collision energy (70 eV) used in electron ionization results in a complex profile of fragmented ions. Therefore, TIC, which includes the entire population of fragment ions within the selected m/z window, is more suitable for peak area calculation than EIC. We found that most RRF values calculated from TIC showed good reproducibility across the concentration range from 5 to 20 µg/mL (Fig. S3).

In LC-MS analysis, employing EIC constructed with predominant fragment ions for calculating RRF values is more advantageous and reliable than using TIC. The electrospray ionization process in LC-MS provides milder ionization conditions, resulting in the molecular ion [M + H]^+^ being the predominant ion within the selected *m/z* window. However, chemicals with structurally susceptible or chemically unstable during ionization produce more fragment even under ESI. In our findings, RRF values calculated from EIC constructed from multiple ions were very similar to that calculated from TIC. EIC also allows for selective monitoring of the characteristic product ions of the target analytes, significantly enhancing the S/N ratio. This enhanced S/N ratio is particularly beneficial when analyzing weak signals or those obscured by overlapping peaks in the TIC (Fig. S4). Specific fragment ions used to construct EICs of each reference standard in positive ion mode and negative ion mode of LC-MS analysis are depicted in Tables S5 and S6, respectively.

In this study, although we established a comprehensive list of reference standards using Tier I chemicals, it does not fully encompass the chemical space of medical device extractables and leachables (E&L). Therefore, we believe it is appropriate to address the inclusion and usage of Tiers II and III with expanded chemical universe coverage in a subsequent manuscript. Additionally, we aim to propose the optimal least number of reference standards that might be required for reliable UF calculation across various device materials.

This study presents a practical and feasible list of reference standards, specifically designed to facilitate the development of semi-quantitative techniques for quantifying medical device E&L. The proposed list of 106 polymer additives as a practical resource for selecting reference standards for NTA of polymeric medical device materials, though it is not intended to be prescriptive. Also, the selected reference standards encompass a broad range of physicochemical and toxicological properties to enable a comprehensive assessment of the detectable range of chemicals in both GC-MS and LC-MS analyses providing additional confidence in the toxicological risk assessment.

Our list of reference standards may also serve as a valuable resource for E&L profiling of medical devices, intended to define the capabilities of GC-MS and LC-MS analytical techniques across a range of polymeric materials used in these devices. Analytical chemistry laboratories can utilize this information to assess their capability to detect and quantify chemicals with a wide range of physicochemical and toxicological properties. Improving the consistency and reliability of analytical chemistry testing may enhance the confidence in, and quality of, toxicological risk assessment, thereby strengthening the overall medical device risk evaluation.

We believe this study represents a significant contribution to the field of E&L analysis for medical devices by establishing the first publicly available list of reference standards for quantitative non-targeted analysis. Led by the FDA, this initiative is intended to foster improved communication and collaboration among stakeholders in the medical device industry by offering a publicly accessible framework to support the development and standardization of analytical methodologies.

## Supplementary information

Supplementary Information

## Data Availability

All data generated or analyzed during this study are included in this published article and its accompanying supplementary information file.

## References

[1] U.S. Food & Drug Administration. 2021 CDRH Annual Report Silver Spring, MD: U.S. FDA; 2022 1/31/2022.

[2] U.S. Food & Drug Administration. 2022 CDRH Annual Report Silver Spring, MD: U.S. FDA; 2023 1/26/2023.

[3] U.S. Food & Drug Administration. 2023 CDRH Annual Report Silver Spring, MD: U.S. FDA; 2024 1/17/2024.

[4] U.S. Food & Drug Administration. Use of International Standard ISO 10993-1, “Biological evaluation of medical devices - Part 1: Evaluation and testing within a risk management process”. In: Center for Devices and Radiological Health, Center for Biologics Evaluation and Research, editors. Silver Spring, MD: U.S. FDA; 2023.

[5] International Organization for Standardization. ISO 10993-18:2020 Biological evaluation of medical devices - Part 18: Chemical characterization of medical device materials within a risk management process. ISO 10993. Geneva, Switzerland: ISO; 2020.

[6] International Organization for Standardization. ISO 10993-12:2021 Biological evaluation of medical devices - Part 12: Sample preparation and reference materials. ISO 10993. Geneva, Switzerland: ISO; 2021.

[7] Ball DJ, Norwood DL, Stults CLM, Nagao LM. Leachables and extractables handbook : safety evaluation, qualification, and best practices applied to inhalation drug products. Hoboken, New Jersey: John Wiley & Sons, Inc.; 2012.

[8] Jenke D. Key Definitions and Concepts, Extractables, and Leachables. Extractables and Leachables: Characterization of Drug Products, Packaging, Manufacturing and Delivery Systems, and Medical Devices. Oxford, UK: John Wiley & Sons, Inc.; 2022. p. 15–72.

[9] Moyer KL, Scull J. Extractables and leachables. Specification of Drug Substances and Products 2014. p. 265–89.

[10] Sussman EM, Oktem B, Isayeva IS, Liu J, Wickramasekara S, Chandrasekar V, et al. Chemical characterization and non-targeted analysis of medical device extracts: a review of current approaches, gaps, and emerging practices. ACS Biomater Sci Eng. 2022;8:939–63. 35171560 10.1021/acsbiomaterials.1c01119

[11] Oberacher H, Arnhard K. Compound identification in forensic toxicological analysis with untargeted LC-MS-based techniques. Bioanalysis. 2015;7:2825–40. 26563687 10.4155/bio.15.193

[12] Pitt JJ. Principles and applications of liquid chromatography-mass spectrometry in clinical biochemistry. Clin Biochem Rev. 2009;30:19–34. 19224008 PMC2643089

[13] Product Quality Research Institute. Safety thresholds and best practices for extractables and leachables in orally inhaled and nasal drug products. In: Norwood DL, editor.: PQRI; 2006. 10.1007/s11095-007-9521-z 18183477

[14] Jenke D, Christiaens P, Beusen JM, Verlinde P, Baeten J. A practical derivation of the uncertainty factor applied to adjust the extractables/leachables analytical evaluation threshold (AET) for response factor variation. PDA J Pharm Sci Technol. 2022;76:178–99. 34782443 10.5731/pdajpst.2021.012692

[15] Jenke D, Odufu A. Utilization of internal standard response factors to estimate the concentration of organic compounds leached from pharmaceutical packaging systems and application of such estimated concentrations to safety assessment. J Chromatogr Sci. 2012;50:206–12. 22337797 10.1093/chromsci/bmr048

[16] Aalizadeh R, Alygizakis NA, Schymanski EL, Krauss M, Schulze T, Ibanez M, et al. Development and Application of Liquid Chromatographic Retention Time Indices in HRMS-Based Suspect and Nontarget Screening. Anal Chem. 2021;93:11601–11. 34382770 10.1021/acs.analchem.1c02348

[17] Knolhoff AM, Premo JH, Fisher CM. A proposed quality control standard mixture and its uses for evaluating nontargeted and suspect screening LC/HR-MS method performance. Anal Chem. 2021;93:1596–603. 33274925 10.1021/acs.analchem.0c04036

[18] Jenke D. Correcting the Analytical Evaluation Threshold (AET) and Reported Extractable’s concentrations for analytical response factor uncertainty associated with chromatographic screening for extractables/leachables. PDA J Pharm Sci Technol. 2020;74:348–58. 32295860 10.5731/pdajpst.2019.010520

[19] United States Environmental Protection Agency. Cheminformatics: U.S. EPA; 2024 [Available from: https://www.epa.gov/chemical-research/cheminformatics.

[20] United Nations. Globally Harmonized System (GHS) hazard classification. U.N.; 2019.

[21] Clean Production Action. GreenScreen® for Safer Chemicals. Clean Production Action: Clean Production Action; 2019.

[22] U. S. Food & Drug Administration, The International Council for Harmonisation of Technical Requirements for Pharmaceuticals for Human Use. Q2(R1) Validation of Analytical Procedures: Text and Methodology Guidance for Industry. Silver Spring, MD: U.S. FDA:ICH; 2005. p. 22.

[23] International Organization for Standardization. ISO/TS 21726:2019 Biological evaluation of medical devices - Application of the threshold of toxicological concern (TTC) for assessing biocompatibility of medical device constituents. ISO/TS 21726 ISO; 2019.

[24] Organisation for Economic Co-operation and Development. OECD QSAR Toolbox [Version 5.4]. OECD; 2023.

[25] Benfenati E, Manganaro A, Gini G, editors. VEGA in Silico platform -Version 1.2.3. CEUR Workshop Proceedings 2013; Turin, Italy: VEGAHUB.

[26] International Organization for Standardization. ISO 10993-11:2017 Biological evaluation of medical devices - Part 11: Tests for systemic toxicity. ISO; 2006.

[27] International Organization for Standardization. ISO 10993-4:2017 Biological evaluation of medical devices - Part 4: Selection of tests for interactions with blood. ISO 10993. Geneva, Switzerland: ISO; 2017.

[28] Bewick V, Cheek L, Ball J. Statistics review 7: Correlation and regression. Crit Care. 2003;7:451–9. 14624685 10.1186/cc2401 PMC374386

[29] Berman JJ. Chapter 4 - Understanding Your Data. In: Berman JJ, editor. Data Simplification. Boston: Morgan Kaufmann; 2016. p. 135-87.

[30] Kruve A, Kaupmees K. Adduct Formation in ESI/MS by mobile phase additives. J Am Soc Mass Spectrom. 2017;28:887–94. 28299714 10.1007/s13361-017-1626-y

[31] Oss M, Kruve A, Herodes K, Leito I. Electrospray ionization efficiency scale of organic compounds. Anal Chem. 2010;82:2865–72. 20218595 10.1021/ac902856t

[32] Malm L, Palm E, Souihi A, Plassmann M, Liigand J, Kruve A. Guide to semi-quantitative non-targeted screening using LC/ESI/HRMS. Molecules. 2021;26:3524. 10.3390/molecules26123524 PMC8228683 34207787

[33] Kruve A. Influence of mobile phase, source parameters and source type on electrospray ionization efficiency in negative ion mode. J Mass Spectrom. 2016;51:596–601. 28239972 10.1002/jms.3790

[34] Rebane R, Kruve A, Liigand J, Liigand P, Gornischeff A, Leito I. Ionization efficiency ladders as tools for choosing ionization mode and solvent in liquid chromatography/mass spectrometry. Rapid Commun Mass Spectrom. 2019;33:1834–43. 31381213 10.1002/rcm.8545

[35] Liigand J, Laaniste A, Kruve A. pH Effects on electrospray ionization efficiency. J Am Soc Mass Spectrom. 2017;28:461–9. 27966175 10.1007/s13361-016-1563-1

[36] Liigand J, Wang T, Kellogg J, Smedsgaard J, Cech N, Kruve A. Quantification for non-targeted LC/MS screening without standard substances. Sci Rep. 2020;10:5808. 32242073 10.1038/s41598-020-62573-z PMC7118164

[37] Cheeseman MA, Machuga EJ, Bailey AB. A tiered approach to threshold of regulation. Food Chem Toxicol. 1999;37:387–412. 10418955 10.1016/s0278-6915(99)00024-1

