# Supplementary material for: Designing a set of reference standards for non-targeted analysis of polymer additives extracted from medical devices
Source: J Expo Sci Environ Epidemiol. 2025 Jul 9;35(6):943–55. doi: 10.1038/s41370-025-00788-w (PMC12583132; doi:10.1038/s41370-025-00788-w)
Supplement: Supplementary file 1 — Supplementary Information [file 41370_2025_788_MOESM1_ESM.docx]

Supplementary Information

**Designing a Set of Reference Standards for Non-Targeted Analysis of Polymer Additives Extracted from Medical Devices**

Byeong Hwa Yun^1^*, Amali Herath^1^, Ying Jin^1^, Jamie Kim^1^, Kerry Belton^2^, Echoleah Rufer^1^, and Omar Rivera Betancourt^1^

^1^Division of Biology, Chemistry and Materials Science (DBCMS), Office of Science and Engineering Laboratories (OSEL), Center for Devices and Radiological Health (CDRH), U.S. Food and Drug Administration, Silver Spring, Maryland 20993

^2^Division of Non-Clinical Science (DNCS), Office of Science (OS), Center for Tobacco Products (CTP), U.S. Food and Drug Administration, Silver Spring, Maryland 20993

**Corresponding author:**

*Byeong Hwa Yun, Ph.D.

Division of Biology, Chemistry and Materials Science

Office of Science and Engineering Laboratories

Center for Devices and Radiological Health

U.S. Food and Drug Administration

Silver Spring, Maryland 20993

Tel: 301-837-7392

Email: [ByeongHwa.Yun@fda.hhs.gov](mailto:ByeongHwa.Yun@fda.hhs.gov)

**Keywords:** extractables, leachables, relative response factor, medical device, biocompatibility, and mass spectrometry

**Summary**

This supplementary information along with the manuscript titled “Designing a Set of Reference Standards for Non-Targeted Analysis of Polymer Additives Extracted from Medical Devices” includes additional information on methods, processed data and tables collected from GC-MS and LC-MS analysis of 106 reference standard materials.

**Table of Contents**

[Equation S1 5](#_Toc179902644)

[Figure S1. 6](#_Toc179902645)

[Figure S2. 17](#_Toc179902646)

[Figure S3.. 18](#_Toc179902647)

[Figure S4. 19](#_Toc179902648)

[Figure S5.. 20](#_Toc179902649)

[Figure S6.. 21](#_Toc179902650)

[Figure S7. 22](#_Toc179902651)

[Figure S8. 23](#_Toc179902652)

[Table S1. 24](#_Toc179902653)

[Table S2. 29](#_Toc179902654)

[Table S3. 30](#_Toc179902655)

[Table S4. 31](#_Toc179902656)

[Table S5. 32](#_Toc179902657)

[Table S6. 35](#_Toc179902658)

[Table S7.. 37](#_Toc179902659)

[Table S8. 39](#_Toc179902660)

[Table S9. 41](#_Toc179902661)

[Table S10. 42](#_Toc179902662)

[Table S11. 43](#_Toc179902663)

[Table S12. 48](#_Toc179902664)

[Table S13. 49](#_Toc179902665)

[References 50](#_Toc179902666)

**Equation S1**

Calculation of AET cited from ISO 10993-18:2020.(1)

$$\mathrm{AET}=(DBT \times\frac{A}{BC})/UF$$

Here,

A is the number of medical devices that underwent extraction to produce the extract;

B is the volume of the extract (measured in mL);

C is the clinical exposure to the medical device (number of devices a user would be exposed to in a day under normal clinical practice);

DBT is the dose-based threshold in μg/day;

UF is an uncertainty factor that could be applied to account for the analytical uncertainty of the screening methods used to estimate extractables’ concentrations in an extract.

# **Figure S1.** Structures of 106 reference standard materials. *RM 84 used for this study was a blend of tri-n-octyl trimellitate (CAS# 89-04-3, MW 546.8) and tri-n-decyl trimellitate (CAS# 4130-35-2, MW 630.9). However, in this study, we monitored tri-n-decyl trimellitate as it was clearly detectable in LC-MS while tri-n-octyl trimellitate showed very weak signal or not detectable at low concentration.

**RM1**

**RM4**

**RM5**

**RM6**

**RM7**

**RM3**

**RM2**

**RM11**

**RM12**

**RM13**

**RM14**

**RM15**

**RM8**

**RM9**

**RM10**

**RM17**

**RM18**

**RM19**

**RM20**

**RM21**

**RM22**

**RM23**

**RM24**

**RM16**

**RM30**

**RM31**

**RM32**

**RM27**

**RM28**

**RM29**

**RM25**

**RM26**

**RM33**

**RM34**

**RM35**

**RM36**

**RM37**

**RM38**

**RM39**

**RM40**

**RM41**

**RM42**

**RM43**

**RM48**

**RM47**

**RM46**

**RM45**

**RM44**

**RM62**

**RM58**

**RM57**

**RM55**

**RM50**

**RM51**

**RM52**

**RM61**

**RM60**

**RM59**

**RM56**

**RM54**

**RM53**

**RM49**

**RM63**

**RM64**

**RM65**

**RM66**

**RM67**

**RM68**

**RM69**

**RM70**

**RM71**

**RM72**

**RM73**

**RM80**

**RM74**

**RM75**

**RM76**

**RM77**

**RM78**

**RM79**

**RM81**

**RM82**

**RM94**

**RM93**

**RM91**

**RM92**

**RM89**

**RM90**

**RM88**

**RM86**

**RM87**

**RM85**

**RM84***

**RM83**

**RM96**

**RM95**

**RM97**

**RM98**

**RM99**

**RM100**

**RM101**

**RM102**

**RM103**

**RM104**

**RM105**

**RM106**

# **Figure S2.** Distribution of physicochemical properties for 106 reference standards tested in this study: (a) molecular weight, (b) boiling point, and (c) logP value.


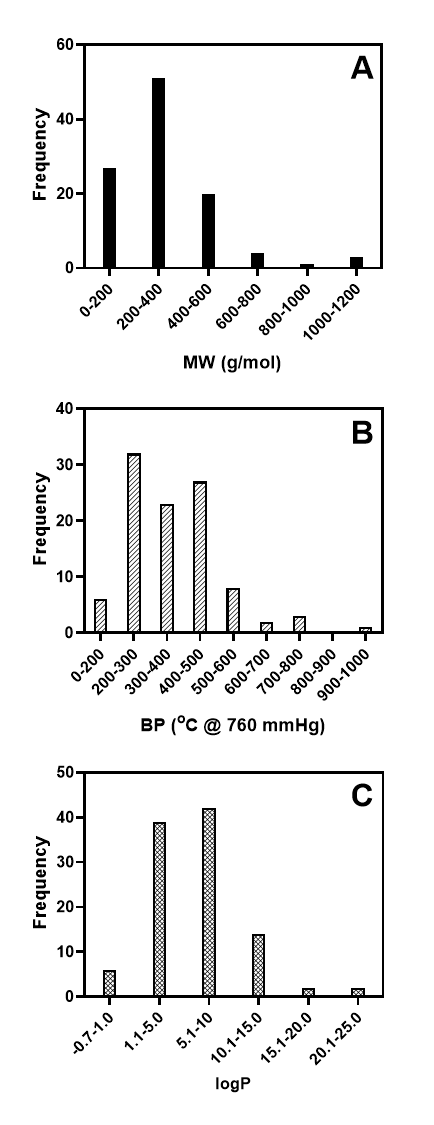
77

# **Figure S3.** Representative total ion chromatograms (TIC) of RM83 measured at three concentrations (5, 10, and 20 µg/mL) in GC-MS analysis. The high energy electron ionization generated complex fragmentation of the parent molecule as shown in the product mass spectrum. Then the sample peak area in TICs is quantitatively correlated to the concentration of sample. Thus, applying TIC for identification and quantitation of sample is more beneficial than using EIC.


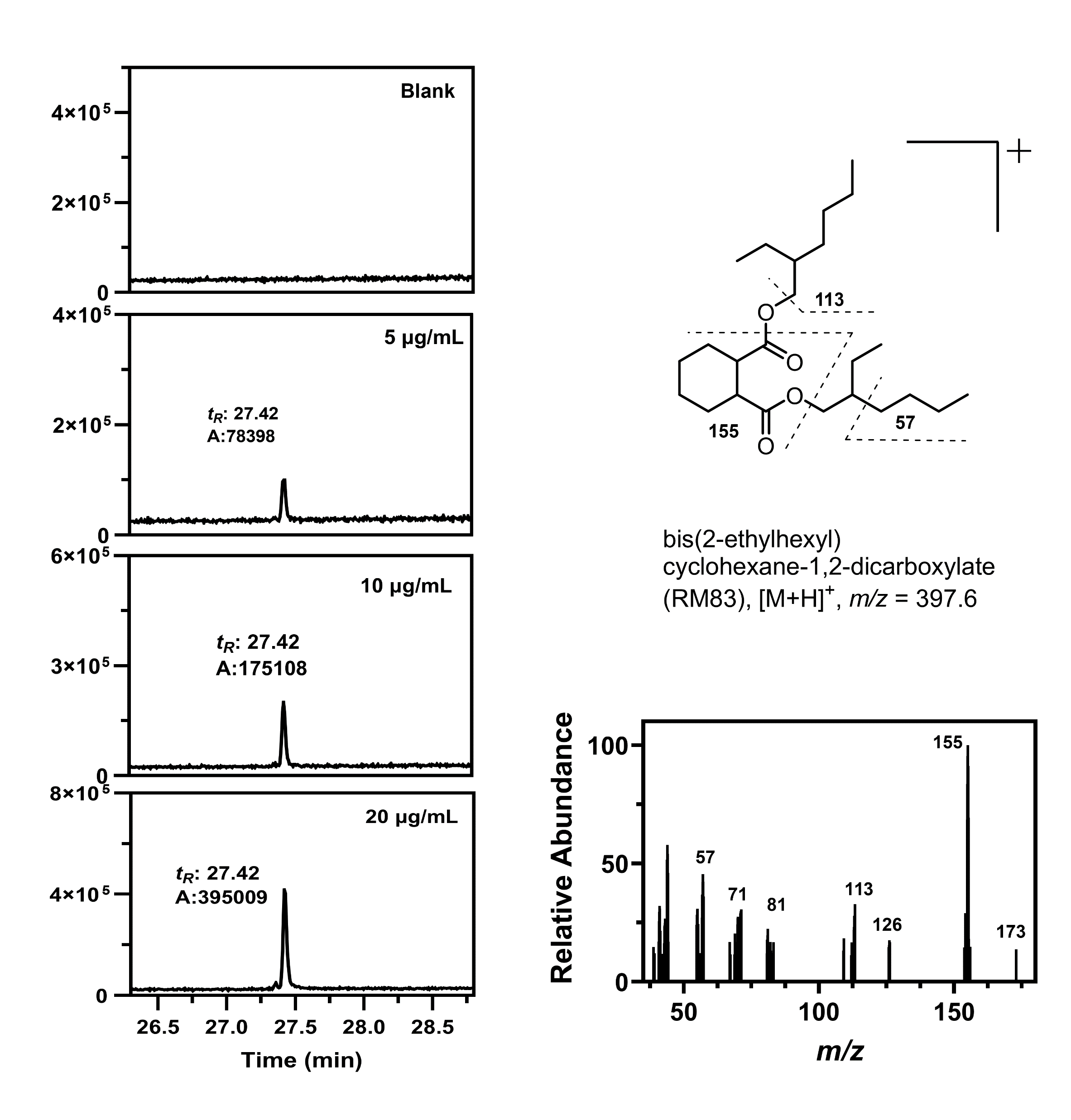


# **Figure S4.** Representative total ion chromatogram (TIC) and extracted ion chromatogram (EIC) of RM15 in LC-MS analysis. RM15 is not detectable in UV detector due to a very weak chromophore and its peak in TIC is overlapped with unknown compound eluted at 38.35 min. However, applying EIC instead of TIC for this sample, it significantly increased the S/N ratio and provided a clean symmetric peak eluted at 38.52 min. Additionally, in the product mass spectrum, the parent ion is typically the most abundant ion because the ESI is a softer ionization method than EI used in GC-MS analysis Applying EIC in LC-MS analysis enhances the quality of identification and quantitation of RM15 in mass spectrometry.


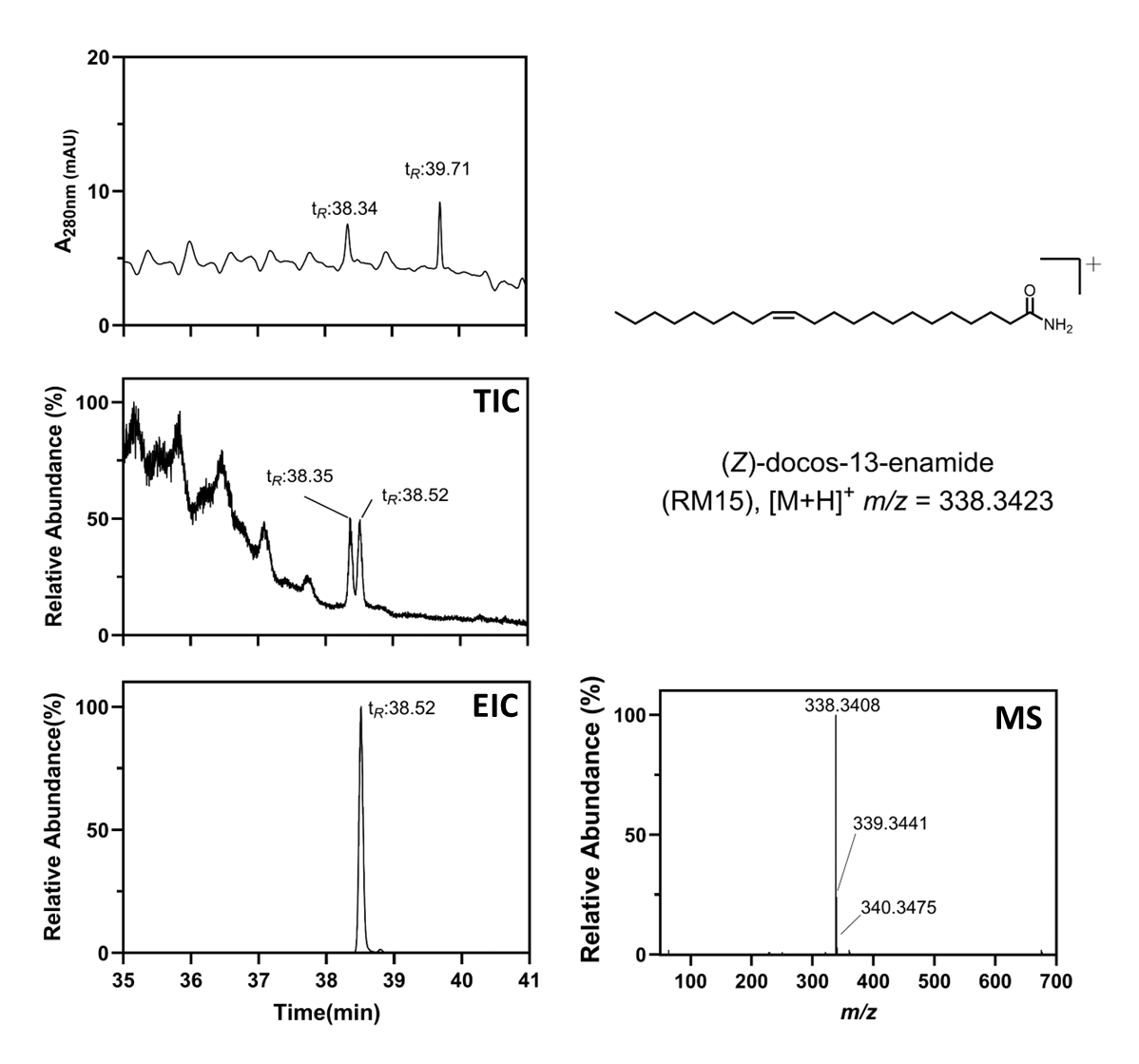


# **Figure S5.** Detail RRF values of reference standards at three concentrations (5, 10, and 20 µg/mL) measured in GC-MS analysis (A), positive ion detection mode (B), and negative ion detection mode (C) in LC-MS analyses.


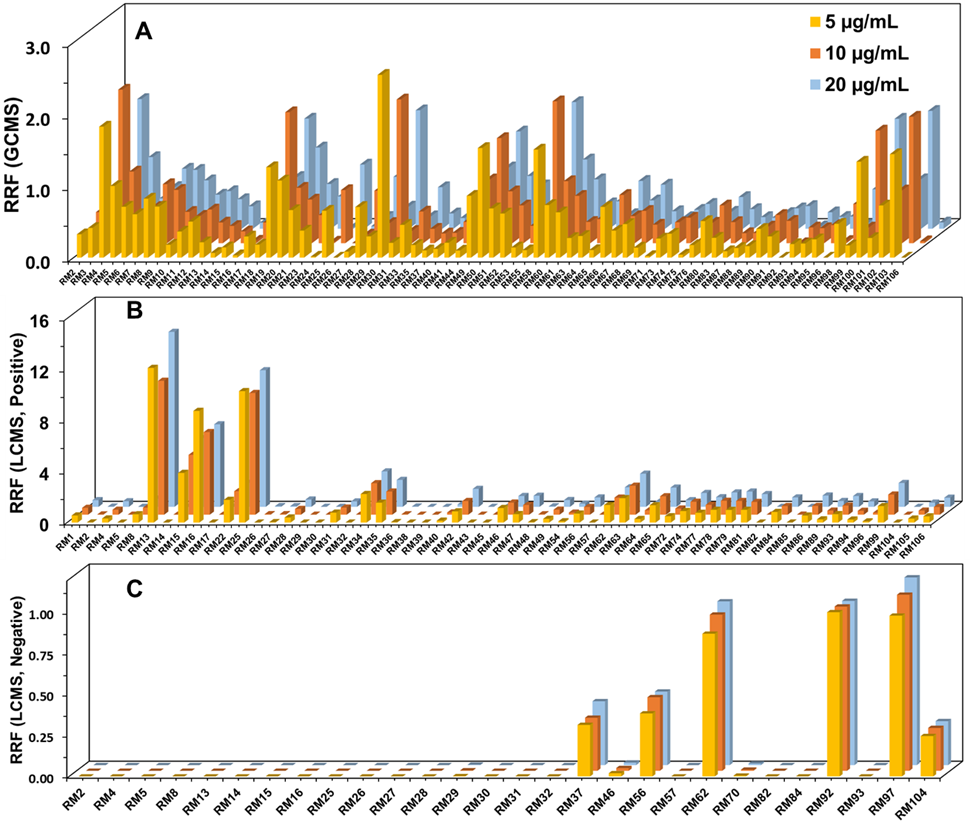


# **Figure S6.** Group II hazards. This figure presents the distribution of various Group II hazard categories: systemic toxicity, skin sensitization, respiratory sensitization, and hemocompatibility. Each category is divided into different levels: Very High (vH), High (H), Moderate (M), Low (L), and Unknown (U). The numbers represent the count and percentage of each level in their respective categories.


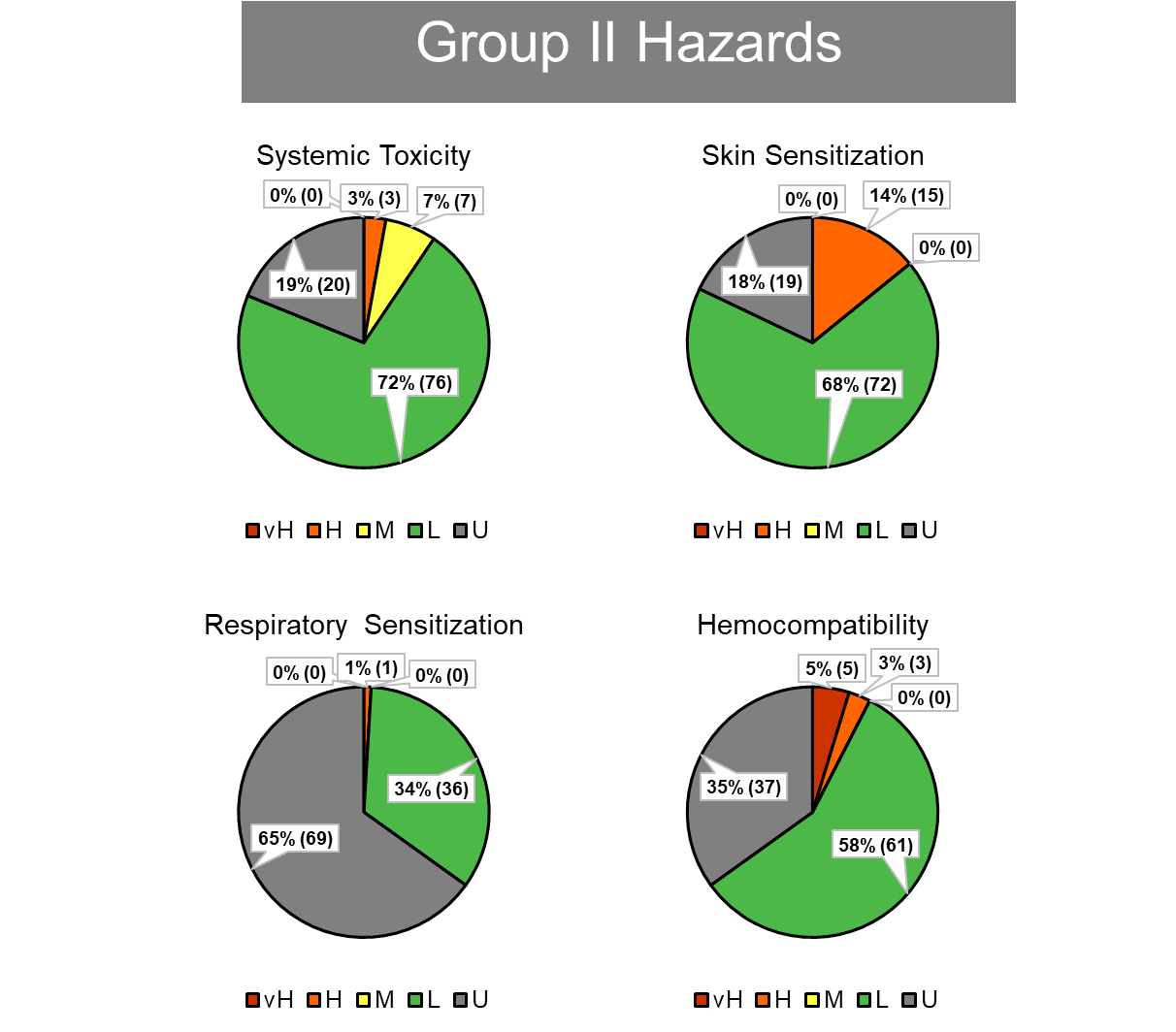


# **Figure S7.** Group III hazards. This figure presents the distribution of various Group III hazard categories: skin irritation, eye irritation, and acute toxicity. Each category is divided into different levels: very high (vH), high (H), moderate (M), low (L), and unknown (U). The numbers represent the count and percentage of each level in their respective categories.


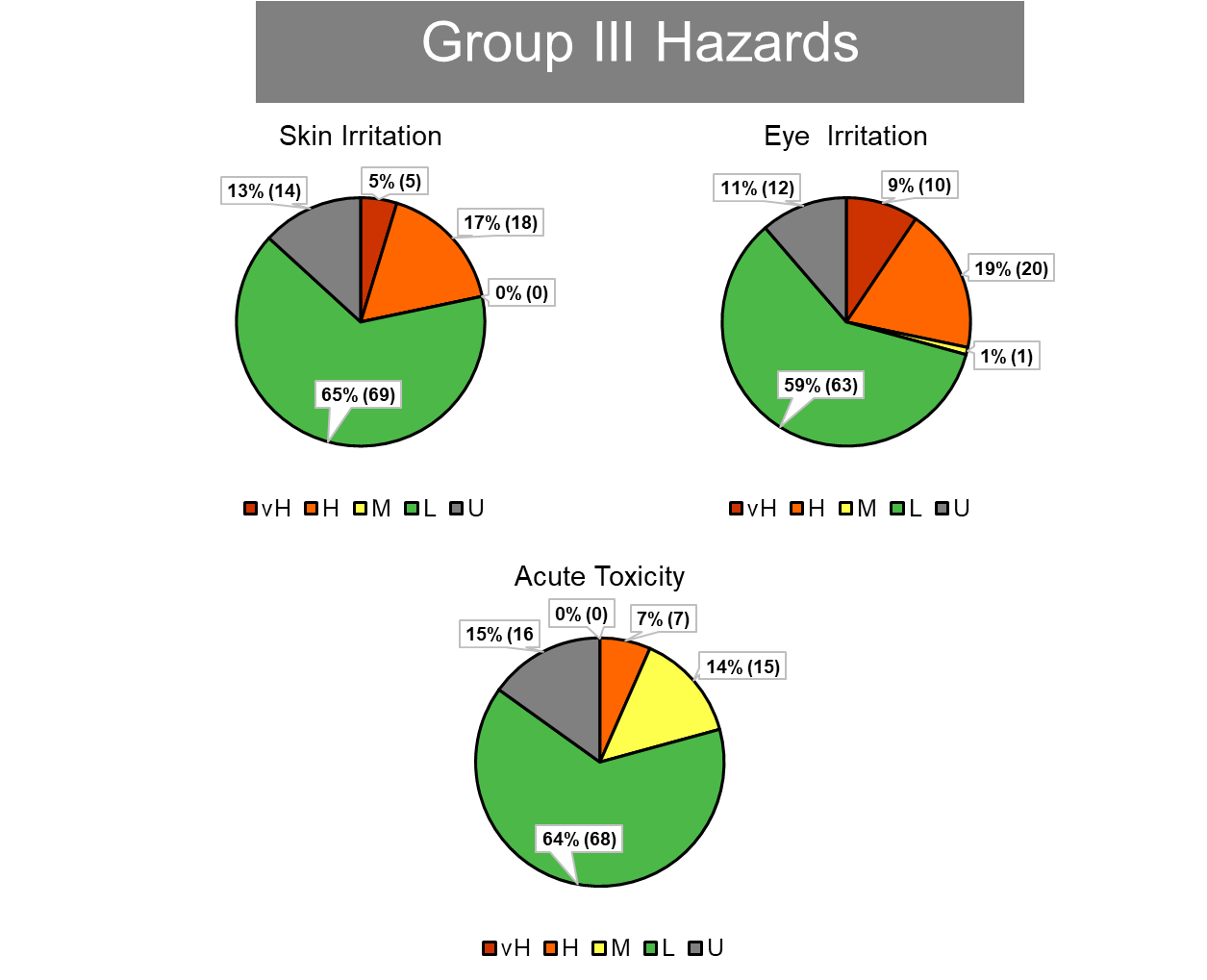


# **Figure S8.** Representative linear regression analysis between four physicochemical properties and RRF values obtained from GCMS data (5 µg/mL)

.


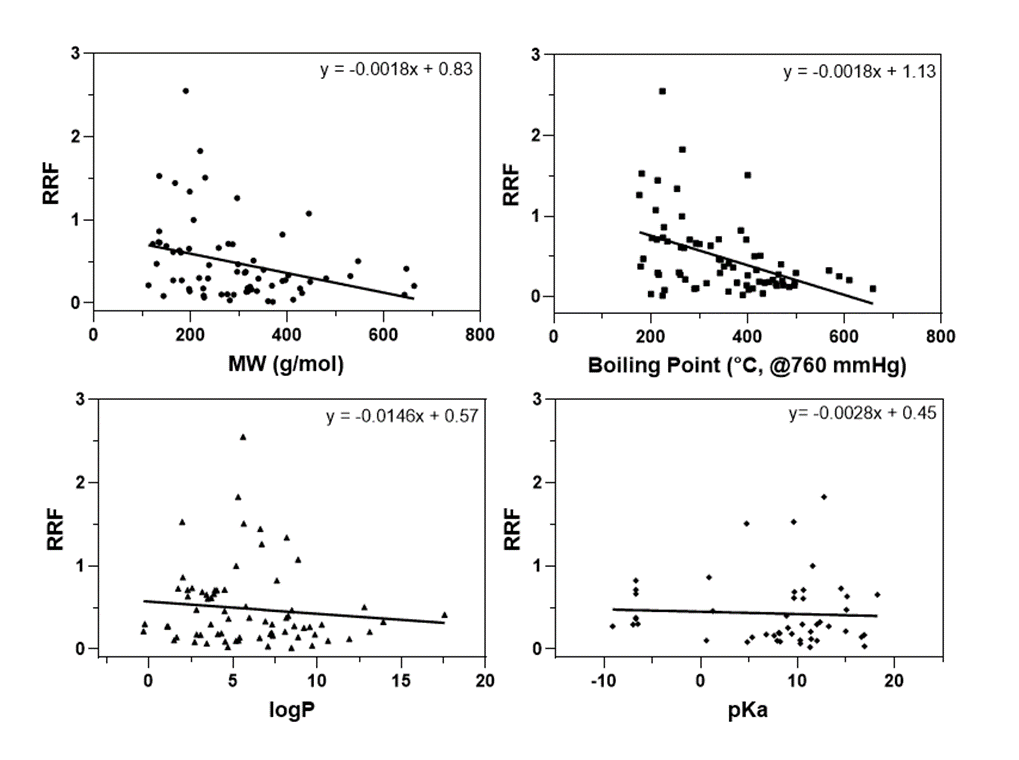


# **Table S1.** Complete list of 106 reference standard materials with their six representative physicochemical properties. This includes DBE, Double Bond Equivalents; Molecular weight (MW, g/mol); Boiling Point (BP, °C at 760 mmHg); pKa, logP, and Refractive Index.

| **Code** | **IUPAC name** | **CAS #** | **Empirical Formula** | **DBE** | **MW** | **BP** | **pKa** | **logP** | **Refractive Index** |
| --- | --- | --- | --- | --- | --- | --- | --- | --- | --- |
| **RM1** | [3-[3-(3,5-di*tert*-butyl-4-hydroxyphenyl)propanoyloxy]-2,2-bis[3-(3,5-di*tert*-butyl-4-hydroxyphenyl)propanoyloxymethyl]propyl] 3-(3,5-di*tert*-butyl-4-hydroxyphenyl)propanoate | 6683-19-8 | C73H108O12 | 20 | 1177.6 | 779.0 | 11.71 | 23.00 | N/A |
| **RM2** | octadecyl 3-(3,5-di*tert*-butyl-4-hydroxyphenyl)propanoate | 2082-79-3 | C35H62O3 | 5 | 530.9 | 568.0 | 12.33 | 13.93 | N/A |
| **RM3** | tris(2,4-di*tert*-butylphenyl) phosphite | 31570-04-4 | C42H63O3P | 11.5 | 646.9 | 361.0 | N/A | 17.56 | N/A |
| **RM4** | 2,6-di*tert*-butyl-4-methylphenol | 128-37-0 | C15H24O | 4 | 220.4 | 265.0 | 12.75 | 5.30 | 1.499 |
| **RM5** | 2,4-di*tert*-butylphenol | 96-76-4 | C14H22O | 3 | 206.3 | 264.0 | 11.56 | 5.19 | 1.499 |
| **RM6** | 2,4-dimethylphenol | 105-67-9 | C8H10O | 4 | 122.2 | 212.0 | 10.60 | 2.30 | 1.540 |
| **RM7** | 2-*tert*-butyl-4-methoxyphenol | 25013-16-5 | C11H16O2 | 4 | 180.2 | 268.0 | 10.58 | 3.50 | 1.491 |
| **RM8** | bis(2-ethylhexyl) benzene-1,2-dicarboxylate | 117-81-7 | C24H38O4 | 6 | 390.6 | 386.0 | -6.70 | 7.60 | 1.486 |
| **RM9** | dibutyl benzene-1,2-dicarboxylate | 84-74-2 | C16H22O4 | 6 | 278.3 | 340.0 | -6.70 | 4.50 | 1.499 |
| **RM10** | diphenyl benzene-1,2-dicarboxylate | 84-62-8 | C20H14O4 | 14 | 318.3 | 497.0 | N/A | 4.10 | 1.616 |
| **RM11** | 2-*O*-benzyl 1-*O*-butyl benzene-1,2-dicarboxylate | 85-68-7 | C19H20O4 | 10 | 312.4 | 370.0 | -6.70 | 4.73 | 1.553 |
| **RM12** | tris(2-ethylhexyl) benzene-1,2,4-tricarboxylate | 3319-31-1 | C33H54O6 | 7 | 546.8 | 414.0 | N/A | 12.80 | 1.488 |
| **RM13** | azepan-2-one | 105-60-2 | C6H11NO | 1.5 | 113.2 | 271.0 | 14.99 | -0.32 | 1.446 |
| **RM14** | 4-[2-(4-hydroxyphenyl)propan-2-yl]phenol | 80-05-7 | C15H16O2 | 8 | 228.3 | 360.0 | 10.29 | 3.43 | 1.599 |
| **RM15** | (*Z*)-docos-13-enamide | 112-84-5 | C22H43NO | 1.5 | 337.6 | 474.0 | 16.61 | 8.87 | 1.469 |
| **RM16** | (*Z*)-octadec-9-enamide | 301-02-0 | C18H35NO | 1.5 | 281.5 | 200.0 | 16.92 | 7.07 | 1.470 |
| **RM17** | butyl octadecanoate | 123-95-5 | C22H44O2 | 1 | 340.6 | 343.0 | -7.00 | 10.27 | 1.448 |
| **RM18** | 2-(benzotriazol-2-yl)-4,6-di*tert*-butylphenol | 3846-71-7 | C20H25N3O | 10 | 323.4 | 444.0 | 9.41 | 7.22 | 1.586 |
| **RM19** | 2,2,4,4,6,6,8,8-octamethyl-1,3,5,7,2,4,6,8-tetraoxatetrasilocane | 556-67-2 | C8H24O4Si4 | 1 | 296.6 | 176.0 | N/A | 6.70 | 1.423 |
| **RM20** | 2,2,4,4,6,6,8,8,10,10,12,12-dodecamethyl-1,3,5,7,9,11-hexaoxa-2,4,6,8,10,12-hexasilacyclododecane | 540-97-6 | C12H36O6Si6 | 1 | 444.9 | 210.0 | N/A | 8.87 | 1.431 |
| **RM21** | bis(2-methylpropyl) hexanedioate | 141-04-8 | C14H26O4 | 2 | 258.4 | 293.0 | -6.70 | 3.85 | 1.439 |
| **RM22** | bis(6-methylheptyl) nonanedioate | 26544-17-2 | C25H48O4 | 2 | 412.7 | NA | N/A | 9.33 | 1.453 |
| **RM23** | dibutyl decanedioate | 109-43-3 | C18H34O4 | 2 | 314.5 | 179.0 | -6.70 | 5.97 | 1.448 |
| **RM24** | 1-*O*-heptyl 6-*O*-nonyl hexanedioate | 68515-75-3 | C22H42O4 | 2 | 370.6 | 224.0 | N/A | 8.47 | 1.452 |
| **RM25** | *N*-benzyl-1-phenylmethanamine | 103-49-1 | C14H15N | 8 | 197.3 | 300.0 | 18.25 | 3.42 | 1.581 |
| **RM26** | benzoic acid | 65-85-0 | C7H6O2 | 5 | 122.1 | 249.0 | 4.00 | 1.89 | 1.564 |
| **RM27** | 2-ethylhexanoic acid | 149-57-5 | C8H16O2 | 1 | 144.2 | 228.0 | 4.82 | 2.72 | 1.435 |
| **RM28** | 1-chloro-4-(4-chlorophenyl)sulfonylbenzene | 80-07-9 | C12H8Cl2O2S | 8 | 287.2 | 397.0 | N/A | 3.90 | 1.610 |
| **RM29** | 2,6-di*tert*-butyl-4-(hydroxymethyl)phenol | 88-26-6 | C15H24O2 | 4 | 236.4 | 214.0 | 12.01 | 3.67 | 1.519 |
| **RM30** | 1,3-di*tert*-butylbenzene | 1014-60-4 | C14H22 | 4 | 190.3 | 223.6 | N/A | 5.59 | 1.478 |
| **RM31** | tris(2,4-di*tert*-butylphenyl) phosphate | 95906-11-9 | C42H63O4P | 12 | 662.9 | 609.8 | N/A | 13.12 | 1.514 |
| **RM32** | hexadecanoic acid | 57-10-3 | C16H32O2 | 1 | 256.4 | 352.0 | 4.95 | 7.17 | 1.454 |
| **RM33** | *N*-(1,3-benzothiazol-2-ylsulfanyl)-2-methylpropan-2-amine | 95-31-8 | C11H14N2S2 | 7.5 | 238.4 | 344.0 | 1.25 | 4.49 | 1.642 |
| **RM34** | 1,2-bis(2-methylphenyl)guanidine | 97-39-2 | C15H17N3 | 9 | 239.3 | 396.0 | 10.32 | 3.77 | 1.585 |
| **RM35** | 1-*N*-phenyl-4-*N*-propan-2-ylbenzene-1,4-diamine | 101-72-4 | C15H18N2 | 8 | 226.3 | 378.0 | 6.76 | 2.83 | 1.634 |
| **RM36** | 1,3,5-tris[(3,5-di*tert*-butyl-4-hydroxyphenyl)methyl]-1,3,5-triazinane-2,4,6-trione | 27676-62-6 | C48H69N3O6 | 16 | 784.1 | NA | 11.45 | 10.34 | 1.567 |
| **RM37** | 5-chloro-2-(2,4-dichlorophenoxy)phenol | 3380-34-5 | C12H7Cl3O2 | 8 | 289.5 | 290.0 | 7.90 | 5.17 | 1.632 |
| **RM38** | zinc;1-oxidopyridin-1-ium-2-thiolate | 13463-41-7 | C10H8N2O2S2Zn | 8 | 317.7 | NA | N/A | 0.90 | N/A |
| **RM39** | dimethyl-octadecyl-(3-trihydroxysilylpropyl)azanium;chloride | 199111-50-7 | C23H52ClNO3Si | **-2** | 454.2 | NA | N/A | N/A | N/A |
| **RM40** | 2,5,7,8-tetramethyl-2-(4,8,12-trimethyltridecyl)-3,4-dihydrochromen-6-ol | 10191-41-0 | C29H50O2 | 5 | 430.7 | 485.9 | 11.40 | 11.90 | 1.495 |
| **RM41** | 2-*tert*-butyl-6-[(3-*tert*-butyl-5-ethyl-2-hydroxyphenyl)methyl]-4-ethylphenol | 88-24-4 | C25H36O2 | 8 | 368.6 | 452.0 | 11.37 | 8.09 | 1.544 |
| **RM42** | 4-(1-phenylethyl)-*N*-[4-(1-phenylethyl)phenyl]aniline | 60160-25-0 | C28H27N | 16 | 377.5 | 288.0 | N/A | 7.64 | 1.626 |
| **RM43** | (piperidine-1-carbothioyltrisulfanyl) piperidine-1-carbodithioate | 120-54-7 | C12H20N2S6 | 4 | 384.7 | 510.0 | 0.85 | 5.50 | 1.730 |
| **RM44** | 2-[2-[3-(3,5-di*tert*-butyl-4-hydroxyphenyl)propanoyloxy]ethylsulfanyl]ethyl 3-(3,5-di*tert*-butyl-4-hydroxyphenyl)propanoate | 41484-35-9 | C38H58O6S | 10 | 642.9 | 659.0 | 12.02 | 10.64 | 1.533 |
| **RM45** | 3,9-bis[2,4-bis(2-phenylpropan-2-yl)phenoxy]-2,4,8,10-tetraoxa-3,9-diphosphaspiro[5.5]undecane | 154862-43-8 | C53H58O6P2 | 25 | 853.0 | 778.0 | N/A | 15.75 | N/A |
| **RM46** | 2-[3,5-bis[2-[3-(3,5-di*tert*-butyl-4-hydroxyphenyl)propanoyloxy]ethyl]-2,4,6-trioxo-1,3,5-triazinan-1-yl]ethyl 3-(3,5-di*tert*-butyl-4-hydroxyphenyl)propanoate | 34137-09-2 | C60H87N3O12 | 19 | 1042.4 | 922.0 | 11.85 | 12.06 | 1.543 |
| **RM47** | *N*,*N*-bis(2-hydroxyethyl)dodecanamide | 120-40-1 | C16H33NO3 | 1 | 287.4 | 443.0 | 14.16 | 3.94 | 1.480 |
| **RM48** | 2,3-dihydroxypropyl octadecanoate | 123-94-4 | C21H42O4 | 1 | 358. 6 | 476.9 | 13.39 | 7.23 | 1.469 |
| **RM49** | 1,3-benzothiazole | 95-16-9 | C7H5NS | 6 | 135.2 | 227.0 | 0.85 | 2.01 | 1.689 |
| **RM50** | *N*,*N*-dimethyl-1-phenylmethanamine | 103-83-3 | C9H13N | 4 | 135.2 | 181.0 | 9.62 | 1.98 | 1.517 |
| **RM51** | 4-*tert*-butylphenol | 98-54-4 | C10H14O | 4 | 150.2 | 234.0 | 9.67 | 3.17 | 1.514 |
| **RM52** | 4-(2-methylbutan-2-yl)phenol | 80-46-6 | C11H16O | 4 | 164.2 | 262.5 | 9.64 | 3.70 | 1.510 |
| **RM53** | 4-[(4-aminophenyl)methyl]aniline | 101-77-9 | C13H14N2 | 8 | 198.3 | 398.0 | 5.32 | 1.64 | 1.660 |
| **RM54** | 6-(dibutylamino)-1*H*-1,3,5-triazine-2,4-dithione | 29529-99-5 | C11H20N4S2 | 4 | 272.4 | 343.7 | 6.06 | 2.12 | 1.622 |
| **RM55** | *N*-(1,3-benzothiazol-2-ylsulfanyl)cyclohexanamine | 95-33-0 | C13H16N2S2 | 7 | 264.4 | 410.4 | 0.59 | 5.32 | 1.665 |
| **RM56** | 3*H*-1,3-benzothiazole-2-thione | 149-30-4 | C7H5NS2 | 6 | 167.3 | 281.3 | 9.80 | 2.41 | 1.757 |
| **RM57** | octadecanoic acid | 57-11-4 | C18H36O2 | 1 | 284.5 | 359.4 | 4.70 | 8.22 | 1.456 |
| **RM58** | 1,4-diphenylbenzene | 92-94-4 | C18H14 | 12 | 230.3 | 400.0 | 4.75 | 5.63 | 1.602 |
| **RM59** | *N*-[2-(octadecanoylamino)ethyl]octadecanamide | 110-30-5 | C38H76N2O2 | 2 | 593.0 | 720.3 | 15.53 | 14.99 | 1.467 |
| **RM60** | 3,4-dimethylbenzaldehyde | 5973-71-7 | C9H10O | 5 | 134.2 | 225.3 | N/A | 2.56 | 1.551 |
| **RM61** | 2,2-dimethyl-*N*-phenylpropanamide | 6625-74-7 | C11H15NO | 5 | 177.2 | 323.5 | 15.12 | 2.31 | 1.545 |
| **RM62** | sodium;1,3,7,9-tetra*tert*-butyl-11-oxido-5*H*-benzo[d][1,3,2]benzodioxaphosphocine 11-oxide | 85209-91-2 | C29H42NaO4P | 9.5 | 508.6 | 510.4 | 2.10 | 0.80 | N/A |
| **RM63** | triethyl phosphate | 78-40-0 | C6H15O4P | 0 | 182.2 | 216.0 | -9.10 | 1.08 | 1.406 |
| **RM64** | 2,3-diacetyloxypropyl acetate | 102-76-1 | C9H14O6 | 3 | 218.2 | 258.0 | -6.50 | -0.24 | 1.435 |
| **RM65** | triethyl 2-hydroxypropane-1,2,3-tricarboxylate | 77-93-0 | C12H20O7 | 3 | 276.3 | 294.0 | 10.28 | 1.49 | 1.462 |
| **RM66** | [2,2,4-trimethyl-3-(2-methylpropanoyloxy)pentyl] 2-methylpropanoate | 6846-50-0 | C16H30O4 | 2 | 286.4 | 280.0 | N/A | 4.04 | 1.441 |
| **RM67** | methyl (*Z*)-octadec-9-enoate | 112-62-9 | C19H36O2 | 2 | 296.5 | 351.4 | N/A | 8.16 | 1.454 |
| **RM68** | propan-2-yl hexadecanoate | 142-91-6 | C19H38O2 | 1 | 298.5 | 340.7 | N/A | 8.49 | 1.443 |
| **RM69** | diphenyl benzene-1,4-dicarboxylate | 1539-04-4 | C20H14O4 | 14 | 318.3 | 496.6 | N/A | 5.39 | 1.616 |
| **RM70** | 6-amino-4-(ethylamino)-1*H*-1,3,5-triazin-2-one | 7313-54-4 | C5H9N5O | 4 | 155.2 | 464.4 | 7.53 | 1.13 | 1.700 |
| **RM71** | tributyl 2-hydroxypropane-1,2,3-tricarboxylate | 77-94-1 | C18H32O7 | 3 | 360. 5 | 389.8 | 11.30 | 4.68 | 1.465 |
| **RM72** | bis(6-methylheptyl) benzene-1,2-dicarboxylate | 27554-26-3 | C24H38O4 | 6 | 390. 6 | 384.9 | N/A | 8.71 | 1.489 |
| **RM73** | bis(2-ethylhexyl) benzene-1,4-dicarboxylate | 6422-86-2 | C24H38O4 | 11 | 390.6 | 400.0 | N/A | 9.55 | 1.489 |
| **RM74** | tributyl 2-acetyloxypropane-1,2,3-tricarboxylate | 77-90-7 | C20H34O8 | 4 | 402.5 | 418.1 | N/A | 6.92 | 1.458 |
| **RM75** | dioctyl nonanedioate | 2064-80-4 | C25H48O4 | 2 | 412.7 | 431.3 | N/A | 9.69 | 1.454 |
| **RM76** | bis(2-ethylhexyl) decanedioate | 122-62-3 | C26H50O4 | 2 | 426.7 | 435.5 | N/A | 9.86 | 1.454 |
| **RM77** | bis(7-methyloctyl) benzene-1,2-dicarboxylate | 28553-12-0 | C26H42O4 | 6 | 418.6 | 435.5 | N/A | 10.14 | 1.489 |
| **RM78** | bis(7-methyloctyl) cyclohexane-1,2-dicarboxylate | 166412-78-8 | C26H48O4 | 3 | 424.7 | 489.6 | N/A | 9.69 | 1.466 |
| **RM79** | bis(8-methylnonyl) hexanedioate | 27178-16-1 | C26H50O4 | 2 | 426.7 | 428.0 | N/A | 10.23 | 1.454 |
| **RM80** | dicyclohexyl benzene-1,2-dicarboxylate | 84-61-7 | C20H26O4 | 8 | 330.4 | 425.8 | N/A | 5.76 | 1.544 |
| **RM81** | trihexyl 2-butanoyloxypropane-1,2,3-tricarboxylate | 82469-79-2 | C28H50O8 | 4 | 514.7 | 522.3 | N/A | 11.17 | 1.462 |
| **RM82** | tris(6-methylheptyl) benzene-1,2,4-tricarboxylate | 27251-75-8 | C33H54O6 | 7 | 546.8 | 585.0 | N/A | 12.80 | 1.488 |
| **RM83** | bis(2-ethylhexyl) cyclohexane-1,2-dicarboxylate | 84-71-9 | C24H44O4 | 3 | 396.6 | 463.9 | N/A | 8.63 | 1.465 |
| **RM84** | Tri(n-octyl, n-decyl) trimellitate | 67989-23-5* | C72H120O12 | 13 | 1177.7 | 583.0 | N/A | 20.82 | N/A |
| **RM85** | 2-benzofuran-1,3-dione | 85-44-9 | C8H4O3 | 7 | 148.1 | 295.0 | 2.97 | 1.60 | 1.613 |
| **RM86** | 2*H*-benzotriazole | 95-14-7 | C6H5N3 | 6 | 119.1 | 359.2 | 1.60 | 1.34 | 1.715 |
| **RM87** | 2-(1,3-benzothiazol-2-yl)phenol | 3411-95-8 | C13H9NOS | 10 | 227.3 | 402.2 | 8.21 | 4.53 | 1.719 |
| **RM88** | 2-*tert*-butyl-6-(5-chlorobenzotriazol-2-yl)-4-methylphenol | 3896-11-5 | C17H18ClN3O | 10 | 315.8 | 460.4 | 9.31±0.48 | 6.58 | 1.628 |
| **RM89** | (2-hydroxy-4-octoxyphenyl)-phenylmethanone | 1843-05-6 | C21H26O3 | 9 | 326.4 | 457.9 | 7.59 | 7.36 | 1.548 |
| **RM90** | 2-(benzotriazol-2-yl)-4,6-bis(2-methylbutan-2-yl)phenol | 25973-55-1 | C22H29N3O | 10 | 351.5 | 469.1 | 8.85 | 8.28 | 1.575 |
| **RM91** | bis(2,2,6,6-tetramethylpiperidin-4-yl) decanedioate | 52829-07-9 | C28H52N2O4 | 4 | 480.7 | 499.8 | 10.49 | 7.32 | 1.498 |
| **RM92** | (2,4-dihydroxyphenyl)-phenylmethanone | 131-56-6 | C13H10O3 | 9 | 214.2 | 409.0 | 7.72 | 3.17 | 1.648 |
| **RM93** | 2-(benzotriazol-2-yl)-4-methylphenol | 2440-22-4 | C13H11N3O | 10 | 225.3 | 423.8 | 8.15 | 4.30 | 1.682 |
| **RM94** | 2-(benzotriazol-2-yl)-4-(2,4,4-trimethylpentan-2-yl)phenol | 3147-75-9 | C20H25N3O | 10 | 323.4 | 471.8 | 8.07 | 7.29 | 1.585 |
| **RM95** | 2-(benzotriazol-2-yl)-4,6-bis(2-phenylpropan-2-yl)phenol | 70321-86-7 | C30H29N3O | 18 | 447.6 | 589.3 | 9.00 | 9.21 | 1.619 |
| **RM96** | imidazolidine-2-thione | 96-45-7 | C3H6N2S | 2 | 102.2 | 148.3 | 15.01 | -0.66 | 1.626 |
| **RM97** | 2,2,3,3,4,4,5,5,6,6,7,7,8,8,9,9,10,10,10-nonadecafluorodecanoic acid | 335-76-2 | C10HF19O2 | 1 | 514.1 | 219.0 | 0.52 | 9.53 | 1.289 |
| **RM98** | 2-ethylhexan-1-ol | 104-76-7 | C8H18O | 0 | 130.2 | 184.0 | 15.06 | 2.82 | 1.426 |
| **RM99** | azacyclotridecan-2-one | 947-04-6 | C12H23NO | 2 | 197.3 | 314.9 | 16.91 | 3.06 | 1.440 |
| **RM100** | tetradecane | 629-59-4 | C14H30 | 0 | 198.4 | 253.9 | N/A | 8.19 | 1.429 |
| **RM101** | 2-hydroxy-2-methyl-1-phenylpropan-1-one | 7473-98-5 | C10H12O2 | 5 | 164.2 | 260.8 | 13.23 | 1.13 | 1.533 |
| **RM102** | 2-phenylpropan-2-ol | 617-94-7 | C9H12O | 4 | 136.2 | 202.0 | 14.49 | 1.73 | 1.518 |
| **RM103** | dodec-1-ene | 112-41-4 | C12H24 | 1 | 168.3 | 213.9 | N/A | 6.62 | 1.431 |
| **RM104** | 2-dodecylbenzenesulfonic acid | 27176-87-0 | C18H30O3S | 4 | 326.5 | 204.5 | 0.70 | 6.78 | 1.510 |
| **RM105** | dodecyl 3-(3-dodecoxy-3-oxopropyl)sulfanylpropanoate | 123-28-4 | C30H58O4S | 2 | 514.8 | 580.8 | N/A | 12.88 | 1.472 |
| **RM106** | 2-(2-ethoxyethoxy)ethanol | 111-90-0 | C6H14O3 | 0 | 134.2 | 202.0 | 14.37 | -0.62 | 1.420 |

*RM 84 used for this test was a blend of tri-n-octyl trimellitate (CAS# 89-04-3, MW 546.8) and tri-n-decyl trimellitate (CAS# 4130-35-2, MW 630.9). However, the CAS No. and combined molecular weight are depicted in this table according to the manufacturer's information.

# **Table S2.** Performance assessment of the analytical GC-MS and LC-MS methods used in this study. The within-day and between-day variations were measured using the signal response of representative reference standards prepared in three concentrations (5,10, and 20 µg/mL) on three days in triplicate.

1. GC-MS method

|  |  | **RM5** | | | **RM33** | | | **RM52** | | | **RM66** | | |
| --- | --- | --- | --- | --- | --- | --- | --- | --- | --- | --- | --- | --- | --- |
|  | µg/mL | **5** | **10** | **20** | **5** | **10** | **20** | **5** | **10** | **20** | **5** | **10** | **20** |
| CV(%)  within-day | *Day 1* | 2.3 | 3.2 | 6.7 | 5.6 | 5.2 | 0.3 | 3.6 | 3.8 | 12.5 | 0.7 | 3.1 | 3.2 |
|  | *Day 2* | 3.7 | 2.6 | 5.4 | 5.6 | 4.5 | 4.5 | 1.8 | 2.6 | 4.1 | 2.3 | 2.3 | 4.7 |
|  | *Day 3* | 4.6 | 2.2 | 10.9 | 4.5 | 1.9 | 1.8 | 9.3 | 5.3 | 0.9 | 4.5 | 3.3 | 1.8 |
| CV(%)  between-day |  | 10.4 | 10.6 | 11.6 | 10.3 | 6.6 | 5.9 | 11.6 | 6.1 | 12.3 | 10.1 | 9.8 | 7.2 |

1. LC-MS method

|  |  | **RM81 (pos)** | | | **RM89 (pos)** | | | **RM92 (neg)** | | |
| --- | --- | --- | --- | --- | --- | --- | --- | --- | --- | --- |
|  | µg/mL | **5** | **10** | **20** | **5** | **10** | **20** | **5** | **10** | **20** |
| CV(%)  within-day | *Day 1* | 0.7 | 1.5 | 0.2 | 3.5 | 5.7 | 7.8 | 7.7 | 7.3 | 5.7 |
|  | *Day 2* | 2.1 | 2.1 | 3.1 | 4.4 | 9.4 | 7.5 | 5.6 | 8.3 | 6.3 |
|  | *Day 3* | 7.6 | 4.6 | 2.8 | 3.2 | 7.8 | 5.2 | 2.6 | 4.5 | 5.5 |
| CV(%)  between-day |  | 9.8 | 9.7 | 8.3 | 16.1 | 14.8 | 15.8 | 10.8 | 10.3 | 10.2 |

# **Table S3.** Limit of detection (LOD) and Limit of quantification (LOQ) of analytical (A) GC-MS and (B) LC-MS methods determined by the calibration curves of representative reference standards used in this study.

A

| *Code* | *LOD*  *(µg/mL)* | *LOQ*  *(µg/mL)* | *Calibration range*  *(µg/mL)* | *Linearity* | *R^2^* |
| --- | --- | --- | --- | --- | --- |
| *RM5* | 0.7 | 2.2 | 2.5-10 | y = 66277x - 76739 | 0.9992 |
| *RM33* | 0.8 | 2.4 | 2.5-20 | y = 12755x - 31758 | 0.9989 |
| *RM52* | 2.1 | 6.4 | 1.0-20 | y = 27301x - 34078 | 0.9961 |
| *RM66* | 1.2 | 3.5 | 1.0-20 | y = 44501x - 50699 | 0.9964 |

B

| *Code* | *LOD*  *(µg/mL)* | *LOQ*  *(µg/mL)* | *Calibration range*  *(µg/mL)* | *Linearity* | *R^2^* |
| --- | --- | --- | --- | --- | --- |
| *RM8 (pos)* | 1.8 | 5.5 | 2.5-20 | y = 30653x + 10939 | 0.9979 |
| *RM81 (pos)* | 2.3 | 7.0 | 2.5-20 | y = 38204x + 14281 | 0.9908 |
| *RM89 (pos)* | 1.4 | 4.2 | 1.0-20 | y = 24963x + 12365 | 0.9967 |
| *RM92 (neg)* | 1.3 | 4.1 | 1.0-15 | y = 25333x + 17892 | 0.9942 |

# **Table S4.** List of vendors and manufacturers of chemicals used in this study.

| Vendor | Corresponding sample Codes |
| --- | --- |
| Sigma  (Saint Louis, MO USA) | RM1, RM2, RM4, RM5, RM8, RM13, RM14, RM15, RM16, RM17, RM19, RM20, RM26, RM27, RM28, RM29, RM30, RM31, RM32, RM34, RM36, RM41, RM47, RM48, RM49, RM50, RM51, RM53, RM56, RM57, RM58, RM59, RM60, RM63, RM64, RM65, RM67, RM75, RM77, RM78, RM79, RM80, RM86, RM87, RM93, RM94, RM97, RM98, RM99, RM100, RM101, RM102, RM103, RM104, RM105, RM106 |
| Accustandard  (New Haven, CT USA) | RM1, RM2, RM3, RM4, RM6, RM7, RM8, RM9, RM10, RM11, RM12, RM14, RM15, RM21, RM22, RM23, RM24, RM25, RM36, RM37, RM38, RM40, RM42, RM43, RM44, RM45, RM46, RM56, RM70, RM71, RM72, RM73, RM74, RM76, RM81, RM82, RM83, RM84, RM85, RM88, RM89, RM90, RM91, RM92, RM93, RM95, RM96 |
| AK Scientific  (Union City, CA USA) | RM18, RM61 |
| Alfa Chemistry  (Holbrook, NY USA) | RM33 |
| TCI America  (Portland, OR USA) | RM35, RM52, RM54, RM55, RM62, RM66, RM68, RM69 |
| Gelest  (Morrisville, PA USA) | RM39 |

# **Table S5.** Specific fragment ions from reference standards were used for positive ion mode of LC-MS analysis to construct extracted ion chromatograms (EIC). The most intense ion from each set of monitored fragments is highlighted in bold.

| **Code** | **IUPAC Name** | **CAS No** | ***m*/*z* used for EIC (Positive Ion Mode)** |  |
| --- | --- | --- | --- | --- |
|  |  |  |  |  |
| **RM1** | [3-[3-(3,5-di*tert*-butyl-4-hydroxyphenyl)propanoyloxy]-2,2-bis[3-(3,5-di*tert*-butyl-4-hydroxyphenyl)propanoyloxymethyl]propyl] 3-(3,5-di*tert*-butyl-4-hydroxyphenyl)propanoate | 6683-19-8 | **1194.8188,** 1195.8221, 1196.8248, 1197.8279 |  |
| **RM2** | octadecyl 3-(3,5-di*tert*-butyl-4-hydroxyphenyl)propanoate | 2082-79-3 | 475.4148, 531.4772, 548.5039, **553.4594**, 572.5041 |  |
| **RM4** | 2,6-di*tert*-butyl-4-methylphenol | 128-37-0 | **219.1745** |  |
| **RM5** | 2,4-di*tert*-butylphenol | 96-76-4 | 207.1743 |  |
| **RM8** | bis(2-ethylhexyl) benzene-1,2-dicarboxylate | 117-81-7 | 149.0235, 391.2839, 392.2873, **413.2661** |  |
| **RM13** | azepan-2-one | 105-60-2 | **114.0917** |  |
| **RM14** | 4-[2-(4-hydroxyphenyl)propan-2-yl]phenol | 80-05-7 | 229.1223 |  |
| **RM15** | (*Z*)-docos-13-enamide | 112-84-5 | **338.3416**, 339.3445 |  |
| **RM16** | (*Z*)-octadec-9-enamide | 301-02-0 | 222.1566, 246.6591, **282.2791**, 283.2825, 370.7710 |  |
| **RM17** | butyl octadecanoate | 123-95-5 | 341.3414 |  |
| **RM22** | bis(6-methylheptyl) nonanedioate | 26544-17-2 | **413.3614,** 414.3650, 430.3884, 435.3435, 436.3469 |  |
| **RM25** | *N*-benzyl-1-phenylmethanamine | 103-49-1 | **198.1273**, 199.1306 |  |
| **RM26** | benzoic acid | 65-85-0 | 123.0441, **243.0437** |  |
| **RM27** | 2-ethylhexanoic acid | 149-57-5 | 145.1223, **265.1218** |  |
| **RM28** | 1-chloro-4-(4-chlorophenyl)sulfonylbenzene | 80-07-9 | 203.9882, 204.9866, 212.9936, 213.9920, **224.5015**, 225.5000, 268.9671, 269.9654, 347.9675, 416.2943 |  |
| **RM29** | 2,6-di*tert*-butyl-4-(hydroxymethyl)phenol | 88-26-6 | 237.1849 |  |
| **RM30** | 1,3-di*tert*-butylbenzene | 1014-60-4 | 191.1794 |  |
| **RM31** | tris(2,4-di*tert*-butylphenyl) phosphate | 95906-11-9 | 663.4525, 664.4561, 680.4790, **685.4344**, 686.4377, 687.4412, 701.4084, 708.5100, 709.5136 |  |
| **RM32** | hexadecanoic acid | 57-10-3 | 257.2475 |  |
| **RM34** | 1,2-bis(2-methylphenyl)guanidine | 97-39-2 | **240.1495**, 241.1524 |  |
| **RM35** | 1-*N*-phenyl-4-N-propan-2-ylbenzene-1,4-diamine | 101-72-4 | 184.0993, **227.1543**, 228.1572 |  |
| **RM36** | 1,3,5-tris[(3,5-di*tert*-butyl-4-hydroxyphenyl)methyl]-1,3,5-triazinane-2,4,6-trione | 27676-62-6 | **219.1744**, 473.2797, 784.5262, 801.5528, 802.5562, 822.4818 |  |
| **RM38** | zinc;1-oxidopyridin-1-ium-2-thiolate | 13463-41-7 | 316.9391 |  |
| **RM39** | dimethyl-octadecyl-(3-trihydroxysilylpropyl)azanium;chloride | 199111-50-7 | 454.3478 |  |
| **RM40** | 2,5,7,8-tetramethyl-2-(4,8,12-trimethyltridecyl)-3,4-dihydrochromen-6-ol | 10191-41-0 | **429.3727**, 430.3788, 431.3837, 445.3678, 452.3617 |  |
| **RM42** | 4-(1-phenylethyl)-*N*-[4-(1-phenylethyl)phenyl]aniline | 60160-25-0 | **378.2211**, 379.2245 |  |
| **RM43** | (piperidine-1-carbothioyltrisulfanyl) piperidine-1-carbodithioate | 120-54-7 | 385.0023 |  |
| **RM45** | 3,9-bis[2,4-bis(2-phenylpropan-2-yl)phenoxy]-2,4,8,10-tetraoxa-3,9-diphosphaspiro[5.5]undecane | 154862-43-8 | 853.3781 |  |
| **RM46** | 2-[3,5-bis[2-[3-(3,5-di*tert*-butyl-4-hydroxyphenyl)propanoyloxy]ethyl]-2,4,6-trioxo-1,3,5-triazinan-1-yl]ethyl 3-(3,5-di*tert*-butyl-4-hydroxyphenyl)propanoate | 34137-09-2 | 1042.6363, 1059.6623, 1060.6657, 1061.6688, **1064.6176**, 1065.6201, 1066.6235, 1087.6929 |  |
| **RM47** | *N*,*N*-bis(2-hydroxyethyl)dodecanamide | 204-393-1 | 204.6301, 225.1433, **288.2533**, 289.2562, 310.2347 |  |
| **RM48** | 2,3-dihydroxypropyl octadecanoate | 123-94-4 | 240.1613, 260.6745, 341.3047, **359.3156**, 360.3187, 378.2886, 381.2972, 382.3004, 443.2677, 449.2847, 557.4429 |  |
| **RM49** | 1,3-benzothiazole | 95-16-9 | **136.0215** |  |
| **RM54** | 6-(dibutylamino)-1*H*-1,3,5-triazine-2,4-dithione | 29529-995 | **273.1198** |  |
| **RM56** | 3*H*-1,3-benzothiazole-2-thione | 149-30-4 | **167.9936**, 183.9884 |  |
| **RM57** | octadecanoic acid | 57-11-4 | 285.2788 |  |
| **RM62** | sodium;1,3,7,9-tetra*tert*-butyl-11-oxido-5*H*-benzo[d][1,3,2]benzodioxaphosphocine 11-oxide | 85209-91-2 | 366.6409, 487.2963, 504.3228, 505.3263, **532.3541**, 533.3575, 560.3855, 648.3230 |  |
| **RM63** | triethyl phosphate | 78-40-0 | 98.9845, 155.0466, **183.0781**, 205.0597 |  |
| **RM64** | 2,3-diacetyloxypropyl acetate | 102-76-1 | 140.0024, 149.5341, 158.5394, 159.0657, 170.0474, 181.0291, 238.0605, **241.0683**, 256.0500, 303.0395, 317.0551 |  |
| **RM65** | triethyl 2-hydroxypropane-1,2,3-tricarboxylate | 77-93-0 | 157.0493, 199.0674, 203.0910, 277.1282, 294.1541, **299.1094**, 300.1128, 322.1852, 575.2297 |  |
| **RM72** | bis(6-methylheptyl) benzene-1,2-dicarboxylate | 27554-26-3 | **391.2839**, 392.2873, 413.2659, 414.2692 |  |
| **RM74** | tributyl 2-acetyloxypropane-1,2,3-tricarboxylate | 77-90-7 | 262.1195, 329.1588, **403.2326**, 404.2353, 425.2136, 426.2171 |  |
| **RM77** | bis(7-methyloctyl) benzene-1,2-dicarboxylate | 28553-12-0 | **419.3156**, 420.3184, 441.2968, 442.3002 |  |
| **RM78** | bis(7-methyloctyl) cyclohexane-1,2-dicarboxylate | 166412-78-8 | **425.3625**, 426.3653, 447.3440, 448.3473 |  |
| **RM79** | bis(8-methylnonyl) hexanedioate | 27178-16-1 | 427.3782, 428.3810, 444.4044, 445.4076, **449.3593**, 450.3629 |  |
| **RM81** | trihexyl 2-butanoyloxypropane-1,2,3-tricarboxylate | 82469-79-2 | **515.3578**, 516.3600, 537.3385, 538.3418 |  |
| **RM82** | tris(6-methylheptyl) benzene-1,2,4-tricarboxylate | 27251-75-5 | 547.3993 |  |
| **RM84** | Tri(n-octyl, n-decyl) trimellitate | 67989-23-5 | **547.3988**, 548.4022, 1115.7722, 1116.7752 |  |
| **RM85** | 2-benzofuran-1,3-dione | 85-44-9 | **149.0232** |  |
| **RM86** | 2*H*-benzotriazole | 95-14-7 | **120.0556** |  |
| **RM89** | (2-hydroxy-4-octoxyphenyl)-phenylmethanone | 1843-05-6 | **327.1955**, 328.1988 |  |
| **RM93** | 2-(benzotriazol-2-yl)-4-methylphenol | 2440-22-4 | **226.0975**, 227.1006 |  |
| **RM94** | 2-(benzotriazol-2-yl)-4-(2,4,4-trimethylpentan-2-yl)phenol | 3147-75-9 | **324.2070,** 325.2098, 445.3152, 462.3418, 467.2971 |  |
| **RM96** | imidazolidine-2-thione | 96-45-7 | **103.0329** |  |
| **RM99** | azacyclotridecan-2-one | 947-04-6 | **198.1847**, 199.1880 |  |
| **RM104** | 2-dodecylbenzenesulfonic acid | 27176-87-0 | 327.1988 |  |
| **RM105** | dodecyl 3-(3-dodecoxy-3-oxopropyl)sulfanylpropanoate | 123-28-4 | 515.4129, **532.4386**, 533.4420, 537.3937, 538.3970 |  |
| **RM106** | 2-(2-ethoxyethoxy)ethanol | 111-90-0 | 135.1016, **157.0835** |  |

# **Table S6.** Specific fragment ions from reference standards were used for negative ion mode of LC-MS analysis to construct extracted ion chromatograms (EIC). The most intense ion from each set of monitored fragments is highlighted in bold.

| **New Code** | **IUPAC Name** | **CAS No** | **m/z used for EIC (Negative Ion Mode)** |  |
| --- | --- | --- | --- | --- |
|  |  |  |  |  |
| **RM2** | octadecyl 3-(3,5-di*tert*-butyl-4-hydroxyphenyl)propanoate | 2082-79-3 | 529.4626 |  |
| **RM4** | 2,6-di*tert*-butyl-4-methylphenol | 128-37-0 | 219.1754 |  |
| **RM5** | 2,4-di*tert*-butylphenol | 96-76-4 | 205.1598 |  |
| **RM8** | bis(2-ethylhexyl) benzene-1,2-dicarboxylate | 117-81-7 | 389.2697 |  |
| **RM13** | azepan-2-one | 105-60-2 | 112.0768 |  |
| **RM14** | 4-[2-(4-hydroxyphenyl)propan-2-yl]phenol | 80-05-7 | 227.1078 |  |
| **RM15** | (*Z*)-docos-13-enamide | 112-84-5 | 336.3272 |  |
| **RM16** | (*Z*)-octadec-9-enamide | 301-02-0 | 280.2646 |  |
| **RM25** | *N*-benzyl-1-phenylmethanamine | 103-49-1 | 196.1132 |  |
| **RM26** | benzoic acid | 65-85-0 | 121.0295 |  |
| **RM27** | 2-ethylhexanoic acid | 149-57-5 | 143.1078 |  |
| **RM28** | 1-chloro-4-(4-chlorophenyl)sulfonylbenzene | 80-07-9 | 285.9616 |  |
| **RM29** | 2,6-di*tert*-butyl-4-(hydroxymethyl)phenol | 88-26-6 | **235.1704** |  |
| **RM30** | 1,3-di*tert*-butylbenzene | 1014-60-4 | 189.1649 |  |
| **RM31** | tris(2,4-di*tert*-butylphenyl) phosphate | 95906-11-9 | 661.4391 |  |
| **RM32** | hexadecanoic acid | 57-10-3 | 255.2330 |  |
| **RM37** | 5-chloro-2-(2,4-dichlorophenoxy)phenol | 3380-34-5 | **286.9439**, 287.9491, 288.9428, 289.9461, 290.9398 |  |
| **RM46** | 2-[3,5-bis[2-[3-(3,5-di*tert*-butyl-4-hydroxyphenyl)propanoyloxy]ethyl]-2,4,6-trioxo-1,3,5-triazinan-1-yl]ethyl 3-(3,5-di*tert*-butyl-4-hydroxyphenyl)propanoate | 34137-09-2 | 1040.6221, 1041.6259, 1042.6292, **1086.6288**, 1087.6318, 1088.6349 |  |
| **RM56** | 3*H*-1,3-benzothiazole-2-thione | 149-30-4 | **165.9791** |  |
| **RM57** | octadecanoic acid | 57-11-4 | 283.2643 |  |
| **RM62** | sodium;1,3,7,9-tetra*tert*-butyl-11-oxido-5*H*-benzo[d][1,3,2]benzodioxaphosphocine 11-oxide | 85209-91-2 | **485.2822**, 486.2852, 487.2885, 553.2699 |  |
| **RM70** | 6-amino-4-(ethylamino)-1*H*-1,3,5-triazin-2-one | 7313-54-4 | **154.0730** |  |
| **RM82** | tris(6-methylheptyl) benzene-1,2,4-tricarboxylate | 27251-75-5 | 545.3848 |  |
| **RM84** | Tri(n-octyl, n-decyl) trimellitate | 67989-23-5 | 1175.8707 |  |
| **RM92** | (2,4-dihydroxyphenyl)-phenylmethanone | 131-56-6 | **213.0556**, 214.0589 |  |
| **RM93** | 2-(benzotriazol-2-yl)-4-methylphenol | 2440-22-4 | 224.0829 |  |
| **RM97** | 2,2,3,3,4,4,5,5,6,6,7,7,8,8,9,9,10,10,10-nonadecafluorodecanoic acid | 335-76-2 | 468.9723, **512.9622**, 513.9659 |  |
| **RM104** | 2-dodecylbenzenesulfonic acid | 27176-87-0 | **325.1840**, 326.1875, 327.1798, 393.1717 |  |

# **Table S7.** Individual hazard rankings.

| **Endpoint** | **Modifications from GreenScreen**® **for Safer Chemicals* v1.4 (2)** |
| --- | --- |
| **Acute toxicity** | None |
| **Carcinogenicity** | Follow GreenScreen® for Safer Chemicals guidance if data are available. If no data are available a prediction may be made according to ISO/TS 21726 (3). Assign moderate (M-pred) if predicted to be carcinogenic based on alerts in any OECD QSAR Profilers (4) or VEGA alerts (5).  OECD QSAR Toolbox v4.6 profilers:  Carcinogenicity (genotoxic and nongenotoxic) alert by ISS  Oncologic primary classification  VEGA alerts:   - Carcinogenicity model (CAESAR)-v.2.1.0 - Carcinogenicity model (ISS)-v.1.0.3 - Carcinogenicity model (IRFMN-ISSCAN-CGX)-v.1.0.2 - Carcinogenicity model (IRFMN- Antares)-v.1.0.2 - Carcinogenicity oral classification model (IRFMN)-v.1.0.1 - Carcinogenicity oral Slope Factor model (IRFMN)-v.1.0.1 - Carcinogenicity inhalation classification model (IRFMN)-v.1.0.1 - Carcinogenicity inhalation Slope Factor model (IRFMN)-v.1.0.1 - Carcinogenicity in male rat (CORAL)-v.1.0.0 - Carcinogenicity in female Rat (CORAL)-v.1.0.0   If no alerts are found, then the substance can be assigned low-predicted (L-pred). If the substance is out of the applicability domain and does not have data, it is assigned U (data gap). |
| **Mutagenicity/ Genotoxicity** | Reliable data on mutagenicity/genotoxicity hold greater weight than predictions and therefore drive the hazard ranking, when available. If data on the substance is not available and predicted to be mutagenic according to method outlined in ISO/TS 21726 Clause 5.2 using two computational models (i.e., statistical, and systems-based) then the substance is assigned moderate, predicted (M-pred)(3). For this manuscript, OECD QSAR Toolbox v4.6 and VEGA were used(4, 5). OECD QSAR Toolbox alerts include:   - DNA binding by OASIS - DNA binding by OECD - Protein binding by OASIS - Protein binding by OECD - In vitro mutagenicity (Ames test) alerts by ISS - In vivo mutagenicity (Micronucleus) alerts by ISS - Protein binding alerts for chromosomal aberration by OASIS - Mutagenicity (Ames test) model for aromatic amines (CONCERT/IRFMN)- v. 1.0.0 - Mutagenicity (Ames test) CONSENSUS model- v. 1.0.4 - Chromosomal aberration model (CORAL)- v. 1.0.1 - In vitro Micronucleus activity (IRFMN-VERMEER)- v. 1.0.1 - In vivo Micronucleus activity (IRFMN)-. 1.0.2   VEGA alerts include:   - Mutagenicity (Ames test) model (CAESAR)- v. 2.1.14 - Mutagenicity (Ames test) model (ISS)- v. 1.0.3 - Mutagenicity (Ames test) model (SarPy-IRFMN)- v. 1.0.8 - Mutagenicity (Ames test) model (KNN-Read-Across)- v. 1.0.1   If no alerts are found using the 2 computational models, then the chemical is assigned low, predicted (L-pred).  Substances for which mutagenicity/genotoxicity cannot be predicted (i.e., they’re outside of the applicability domain) in both types of in silico models and toxicological data are insufficient they should be classified as “U”. |
| **Reproductive/ Developmental Toxicity (DART)** | If chemical-specific data are not available to assign DART, then structural alerts were used to predict DART risk using OECD QSAR toolbox v4.6 and VEGA. Any alerts for the following profilers were considered as Moderate-predicted (M-pred) for both developmental and reproductive toxicity:  OECD QSAR profilers:   - Estrogen receptor binding - rtER Expert System (USEPA) - DART Scheme - VEGA alerts: - Developmental Toxicity model (CAESAR)- v. 2.1.8 - Developmental/Reproductive Toxicity library (PG)- v. 2.1.8   If no alerts were present for both profilers, then DART is considered low-predicted (L-pred). If predictions are unable to be made, then it was considered U (data gap). |
| **Endocrine disruption** | Substances with known endocrine disrupting effects were classified according to the toxicity observed (e.g., DART, carcinogenicity, or systemic toxicity), but endocrine disruption was not considered as a separate endpoint since it is not a separate endpoint considered for medical devices according to FDA Biocompatibility Guidance^1^. |
| **Repeated Dose Systemic Toxicity** | None |
| **Skin Sensitization** | None |
| **Respiratory Sensitization** | None |
| **Hemo-compatibility** | No methods are provided in Green Screen for this endpoint. This endpoint is based on ISO 10993-4 and literature search for a history of hemocompatibility, including hemolysis, thrombogenicity, and complement activation (2). If risks for hemocompatibility are found, rate the chemical vH or H depending on severity. If no data are found rate it as L. |
| **Material-Mediated Pyrogenicity** | No methods are provided in Green Screen ® for Safer Chemicals for pyrogenicity. This endpoint is based on a literature search for a history of material-mediated pyrogenicity being associated with the substance. Most are laid out in ISO 10993-11, Annex G. If risks for pyrogenicity are found, rate the chemical H. If no data are found rate it as L. |
| **Skin Irritation** | None |
| **Eye Irritation** | None |

*GreenScreen® for Safer Chemicals is a registered trademark of Clean Production Action. All rights reserved.

# **Table S8.** Databases used for hazard data searching**.**

| Database | Description | URL |
| --- | --- | --- |
| eChemPortal | Provides access to information on the properties of chemicals. | [eChemPortal](https://www.echemportal.org/echemportal/) |
| Austrian Agency for Health and Food Safety (AGES) | Responsible for chemical assessment, providing toxicological data and risk assessments. | [AGES](https://www.ages.at/en/) |
| Integrated Risk Information System (IRIS) | EPA's database of human health effects that may result from exposure to various substances in the environment. | [IRIS](https://www.epa.gov/iris) |
| Chemical Carcinogenesis Research Information System (CCRIS) | Contains carcinogenicity, mutagenicity, tumor promotion, and tumor inhibition test results for over 8,000 chemicals. | [CCRIS](https://www.nlm.nih.gov/toxnet/Accessing_CCRIS_Content_from_PubChem.html) |
| International Agency for Research on Cancer (IARC) | Produces scientific evaluations of the carcinogenicity of chemicals and physical agents. | [IARC](https://www.iarc.who.int/) |
| European Chemicals Agency (ECHA) | Provides information on chemicals, including toxicological and ecotoxicological data. | [ECHA](https://echa.europa.eu/fr/home) |
| National Toxicology Program (NTP) | Conducts toxicological research and testing to generate information on substances in the environment. | [NTP](https://ntp.niehs.nih.gov/) |
| OECD’s High Production Volume (HPV) Chemicals Programme | An initiative that focuses on the evaluation of chemicals produced or imported at high volumes. It aims to improve our understanding of these chemicals by assessing their potential health and environmental risks. | [OECD](https://hpvchemicals.oecd.org/ui/Default.aspx) |
| CompTox Chemistry Dashboard | Provides access to data on a broad array of chemicals, including information on toxicity and exposure amassed from a variety of sources. | [CompTox](https://cfpub.epa.gov/si/si_public_record_report.cfm?dirEntryId=337501) |
| Office of Environmental Health Hazard Assessment (OEHHA) | California agency assessing health risks posed by environmental contaminants. | [OEHHA](https://oehha.ca.gov/library/chemical-databases) |
| 3E Exchange's substance database | The 3E Exchange's substance database offers key details on chemicals, aiding in effective chemical management and compliance. | [3E Exchange](https://exchange.3eco.com/Substances/Search) |
| US EPA ChemInformatics | The US EPA ChemInformatics tool is a suite of analysis modules developed to enhance chemical safety decision-making, providing high-quality chemical structures, physicochemical properties, and predictive modeling tools. | [US EPA ChemInformatics](https://www.epa.gov/comptox-tools/cheminformatics#:~:text=Cheminformatics%20analysis%20modules%20provide%20information,and%20appropriately%20linked%20toxicity%20data.) |
| PubMed | Compilation of citations for biomedical literature. | [PubMed](https://pubmed.ncbi.nlm.nih.gov/) |
| Google Scholar | A tool used to broadly search scholarly literature. | [Google Scholar](https://scholar.google.com/) |

# **Table S9.** Endpoint groupings.

| **Hazard Endpoint Group** | **Endpoints** | **Possible rankings** |
| --- | --- | --- |
| Group I - high weight in overall rankings | Carcinogenicity  Mutagenicity/Genotoxicity  Reproductive  Developmental | H, M, L, U |
| Group II – moderate weight overall rankings | Systemic toxicity (repeated exposure)  Skin sensitization  Respiratory sensitization  Hemocompatibility  Pyrogenicity | vH, H, M, L, U |
| Group III – lowest weight in overall rankings | Acute toxicity  Systemic toxicity (single exposure)  Skin irritation  Eye irritation | vH, H, M, L, U |

# **Table S10.** Overall hazard ranking criteria.

| **Hazard Rank** | **Criteria** | **Instruction** |
| --- | --- | --- |
| 1 – High | 1. H for any Group I hazard endpoint OR 2. vH for any Group II   Note: Predictive data may be used to assign rankings for Group 1 hazard endpoints. Mutagenicity/genotoxicity must have appropriate toxicological data or be predicted. If there are no data for the chemical and it cannot be predicted the chemical is assigned U – Unknown. | If either are true, assign ranking of 1 – High. If mutagenicity/ genotoxicity lacks data or cannot be predicted, then this chemical is assigned as U - Unknown.  If none of the above are true, move to next row. |
| 2 – Moderate | 1. M for any Group I OR 2. H for any Group II OR 3. vH for any Group III OR 4. H for Group III Acute Tox   Note: Predictive data may be used for Group 1 hazards. | If any statement is true, assign ranking of 2 - Moderate.  If not, move to next row. |
| 3 – Low with uncertainty | 1. L Group I + M Group II + L or M or H Group III OR 2. L Group I + L Group II + L or M or H Group III   Note: Predictive data may be used for Group 1 hazards. If systemic repeat tox is DG, efforts should be made to use read across to predict systemic repeat toxicity. | If either statement is true, assign ranking of 3 – Low with uncertainty.  If not, move to next row. |
| 4 – Low | 1. L Group I + L Group II + L Group III + No DG for Group I endpoints + Not DG for Group II Skin sensitization and Systemic repeat dose   Note: Predictive data may not be used to assign Low with confidence rankings. Assessment based on read across is acceptable for Low with confidence ratings. | If true, assign ranking of 4 – Low with confidence.  If not, move to next row. |
| U – Unknown | 1. Group I (carcinogenicity or mutagenicity/genotoxicity) is not known, cannot be predicted or unspecified due to insufficient data. | Assign as U - unknown. |

# **Table S11.** Hazard rankings.

| **Full name** | **CAS #** | **Carc** | **Mut** | **Repro** | **Dev** | **Systemic Tox repeat** | **SnS** | **SnR** | **Hemo** | **Pyro** | **IrS** | **IrE** | **Acute Tox** | **Overall** |
| --- | --- | --- | --- | --- | --- | --- | --- | --- | --- | --- | --- | --- | --- | --- |
| Pentaerythritol tetrakis(3,5-di-tert-butyl-4-hydroxyhydrocinnamate) | 6683-19-8 | L | L-pred | L-pred | L-pred | U | U | U | L | L | H | U | M | 3 |
| Octadecyl 3-(3,5-di-tert-butyl-4-hydroxyphenyl)propionate | 2082-79-3 | L | L | L | L | L | L | U | L | L | L | L | L | 4 |
| Tris(2,4-di-tert-butylphenyl) phosphite | 31570-04-4 | L | L | L | L | L | L | U | L | L | L | L | L | 4 |
| 2,6-Di-t-butyl-4- methylphenol | 128-37-0 | L | L | L | L | L | L | U | U | L | L | L | L | 4 |
| 2,4-Di-t-butyl phenol | 96-76-4 | L | L | L | L | L | L | L | H | L | H | vH | L | 2 |
| 2,4-Dimethylphenol | 105-67-9 | M-pred | M-pred | L-pred | L-pred | H | H | U | vH | L | vH | vH | H | 2 |
| Butylated hydroxyanisole | 25013-16-5 | M | M-pred | L-pred | L-pred | U | U | U | H | L | U | H | U | 2 |
| Bis(2-ethylhexyl) Phthalate | 117-81-7 | L | L | H | L | L | L | L | L | L | L | L | L | 1 |
| Dibutyl phthalate | 84-74-2 | L | L | H | L | L | L | L | L | L | L | L | L | 1 |
| Diphenyl phthalate | 84-62-8 | M-pred | M-pred | L-pred | L-pred | U | U | U | U | L | H | H | U | 2 |
| Benzyl butyl phthalate | 85-68-7 | L | L | H | L | L | L | L | L | L | L | L | L | 1 |
| Tris(2-ethylhexyl) Trimellitate | 3319-31-1 | L | L | L | L | L | L | L | L | L | L | L | L | 4 |
| Caprolactam | 105-60-2 | L | L | L | L | L | L | L | L | L | H | H | M | 3 |
| 4,4′-(Propane-2,2-diyl)diphenol | 80-05-7 | L | L | H | L | L | H | L | L | L | L | vH | L | 1 |
| cis-13-Docosenoamide | 112-84-5 | M-pred | L | L | L | L | L | U | L | L | L | L | L | 2 |
| Cis-9-Octadecanoamide | 301-02-0 | M-pred | L-pred | L-pred | L-pred | U | U | U | U | L | U | U | U | 2 |
| butyl-octadecanoate | 123-95-5 | L | L | L | L | L | L | L | L | L | L | L | L | 4 |
| 2-(2'-HYDROXY-3',5'-DI-TERT-BUTYLPHENYL)BENZOTRIAZOLE | 3846-71-7 | M | L | M | L | M | L | U | vH | L | L | L | L | 2 |
| Octamethyl-1,3,5,7,2,4,6,8-tetroxatetrasilocane | 556-67-2 | L | L | M | L | L | L | L | L | L | L | L | L | 2 |
| Dodecamethylcyclohexasiloxane | 540-97-6 | M-pred | L | L | L | L | L | U | L | L | L | L | L | 2 |
| Diisobutyl adipate | 141-04-8 | L | L | L | L | L | L | L | L | L | L | L | L | 4 |
| Diisooctyl azelate | 26544-17-2 | L | L | L | L | L | L | L | U | L | L | L | L | 4 |
| Dibutyl sebacate | 109-43-3 | L-pred | L | L | L | L | L | L | L | L | L | L | L | 3 |
| Di(n-heptyl, n-nonyl) adipate | 68515-75-3 | L | L | L | L | L | L | L | U | L | L | L | L | 4 |
| Irganox® E 201 | 10191-41-0 | L | L | L | L | L | H | U | L | L | L | L | L | 2 |
| Irganox 3114 | 27676-62-6 | M-pred | M-pred | L | L | L | L | U | L | L | L | L | L | 2 |
| Tinuvin® PED | 2440-22-4 | L | L | L | L | L | H | U | L | L | L | L | L | 2 |
| Dibenzylamine | 103-49-1 | M-pred | L | L-pred | L-pred | U | L | U | U | L | vH | vH | M | 2 |
| Benzoic acid | 65-85-0 | L | L | L | L | H | L | U | U | L | H | H | L | 2 |
| 2-mercaptobenzothiazole | 149-30-4 | H | L | L | L | L | H | U | L | L | L | L | L | 1 |
| 2-Ethylhexanoic acid | 149-57-5 | L | L | M | H | L | L | L | L | L | L | L | L | 1 |
| Bis(4-chlorophenyl) sulfone | 80-07-9 | L | L | L | L | L | L | U | L | L | H | L | L | 3 |
| 3,5-Di-tert-butyl-4-hydroxybenzyl alcohol | 88-26-6 | L-pred | M-pred | L-pred | L-pred | U | L | U | U | L | L | L | L | 2 |
| 1,3-Di-tert-butylbenzene | 1014-60-4 | M-pred | L-pred | L-pred | L-pred | U | U | U | U | L | U | U | U | 2 |
| Stearic Acid | 57-11-4 | L | L-pred | L-pred | L-pred | U | U | U | L | L | U | U | H | 3 |
| Tris(2,4-di-tert-butylphenyl)phosphate | 95906-11-9 | M-pred | L-pred | L-pred | L-pred | U | U | U | U | L | U | U | U | 2 |
| Palmitic acid | 57-10-3 | M-pred | L | L | L | L | L | U | L | L | L | L | L | 2 |
| N-(1,1-Dimethylethyl)-2-benzothiazolesulfenamide | 95-31-8 | M-pred | L | L | L | L | H | U | vH | L | L | L | L | 2 |
| 1,3-Di-o-tolylguanidine | 97-39-2 | M-pred | M-pred | L | L | L | L | U | L | L | L | L | H | 2 |
| N-Isopropyl-N'-phenyl-p-phenylenediamine | 101-72-4 | M-pred | L | L | L | L | H | U | vH | L | L | L | M | 2 |
| 5-Chloro-2-(2,4-dichlorophenoxy)phenol | 3380-34-5 | L | L | L | L | L | L | U | U | L | H | H | L | 3 |
| 1-Hydroxypyridine-2-thione zinc salt | 13463-41-7 | L | L | L | L | L | L | L | U | L | L | vH | H | 3 |
| 3-(trihydroxysilyl)propyldimethyloctadecyl ammonium chloride | 199111-50-7 | M-pred | L-pred | L-pred | L-pred | U | U | U | U | L | U | U | U | 2 |
| 2,2'-Methylene-bis(4-ethyl-6-tert-butylphenol) | 88-24-4 | M-pred | L | L | L | L | L | U | vH | L | L | L | L | 2 |
| 4-(1-Phenylethyl)-N-[4-(1-phenylethyl)phenyl]aniline | 60160-25-0 | M-pred | M-pred | L-pred | L-pred | U | U | U | U | L | U | U | U | 2 |
| Piperidine, 1,1'-(tetrathiodicarbonothioyl)-bis- | 120-54-7 | L-pred | M-pred | L | L | L | H | U | L | L | U | U | U | 2 |
| Thiodiethylene glycol bis(3,5-di-tert-butyl-4-hydroxyhydrocinnamate) | 41484-35-9 | L-pred | L | L | L | L | L | U | L | L | L | L | L | 3 |
| 3,9-bis[2,4-bis(2-Phenylpropan-2-yl)phenoxy]-2,4,8,10-tetraoxa-3,9-diphosphaspiro[5,5]undecane | 154862-43-8 | M-pred | L-pred | L-pred | L-pred | U | U | U | U | L | H | H | U | 2 |
| 3,5-Di-tert-butyl-4-hydroxyhydrocinnamic triester with 1,3,5-tris[2-Hydroxyethyl]-s-triazine-2,4,6[1H,3H,5H]-trione | 34137-09-2 | L | L | L | L | L | L | L | U | L | L | L | L | 4 |
| lauric acid diethanolamide | 120-40-1 | L | L | L | L | L | L | U | L | L | H | H | L | 3 |
| 1-Stearoyl-rac-glycerol | 123-94-4 | L | L | L | L | L | L | L | L | L | L | L | L | 4 |
| 1,3-benzothiazole | 95-16-9 | M-pred | L | L-pred | L-pred | M | L | U | U | L | L | H | H | 2 |
| N,N-dimethyl-1-phenylmethanamine | 103-83-3 | M-pred | L | L-pred | L-pred | L | L | U | L | L | vH | L | M | 2 |
| 4-tert-butylphenol | 98-54-4 | L | L | M | L | L | L | L | L | L | H | H | L | 2 |
| 4-(2-methylbutan-2-yl)phenol | 80-46-6 | L | L | L | L | L | H | L | L | L | vH | vH | L | 2 |
| 4-[(4-aminophenyl)methyl]aniline | 101-77-9 | H | M | L-pred | L-pred | M | H | U | L | L | L | L | H | 1 |
| 6-(dibutylamino)-1H-1,3,5-triazine-2,4-dithione | 29529-99-5 | L-pred | L-pred | L-pred | L-pred | L | H | U | U | L | L | vH | L | 2 |
| N-Cyclohexyl-2-benzothiazolyl sulfenamide | 95-33-0 | L | L | L | L | L | H | U | L | L | L | L | L | 2 |
| 1,4-Diphenylbenzene | 92-94-4 | M-pred | M-pred | L-pred | L-pred | U | U | U | L | L | H | H | L | 2 |
| N-[2-(octadecanoylamino)ethyl] octadecanamide | 110-30-5 | L | L | L | L | L | L | U | U | L | L | L | L | 4 |
| 3,4-Dimethylbenzaldehyde | 5973-71-7 | M-pred | L | L | L-pred | L | U | U | L | L | H | H | L | 2 |
| N-Phenylpivalamide | 6625-74-7 | M-pred | L-pred | L-pred | L-pred | U | U | U | U | L | U | U | U | 2 |
| 2,2'-Methylenbis(4,6-di-tert-butylphenyl)phosphate Sodium Salt | 85209-91-2 | L-pred | L | L | L | L | L | U | L | L | L | L | M | 3 |
| triethyl phosphate | 78-40-0 | L | L | L | L | L | L | L | L | L | L | H | M | 3 |
| 1,2,3-propanetriol triacetate | 102-76-1 | L | L | L | L | L | L | L | L | L | L | L | L | 4 |
| triethyl 2-hydroxypropane-1,2,3-tricarboxylate | 77-93-0 | L | L | L | L | L | L | L | L | L | L | L | L | 4 |
| [2,2,4-trimethyl-3-(2-methylpropanoyloxy)pentyl] 2-methylpropanoate | 6846-50-0 | L | L | L | M | L | L | L | L | L | L | L | L | 2 |
| methyl (Z)-octadec-9-enoate | 112-62-9 | M-pred | L-pred | L-pred | L-pred | U | U | U | U | L | U | H | U | 2 |
| propan-2-yl hexadecanoate | 142-91-6 | L | L | L | L | L | L | L | U | L | L | L | L | 4 |
| diphenyl benzene-1,4-dicarboxylate | 1539-04-4 | M-pred | L-pred | L-pred | L-pred | U | U | U | U | L | U | U | U | 2 |
| bis(2-ethylhexyl) succinate | 7313-54-4 | L-pred | M-pred | L-pred | L-pred | U | U | U | U | L | U | U | U | 2 |
| tributyl 2-hydroxypropane-1,2,3-tricarboxylate | 77-94-1 | L | L | L | L | L | L | U | L | L | L | L | L | 4 |
| bis(6-methylheptyl) benzene-1,2-dicarboxylate | 27554-26-3 | L | L | H | L | L | L | U | L | L | L | L | L | 1 |
| Bis 2-ethylhexyl terephthalate | 6422-86-2 | L | L | L | L | L | L | U | L | L | L | L | L | 4 |
| ATBC Tributyl O-acetylcitrate | 77-90-7 | L | L | L | L | L | L | U | L | L | L | L | L | 4 |
| nonanedioic acid, dioctyl ester, dioctyl azelate | 2064-80-4 | L-pred | L-pred | L-pred | L-pred | U | U | U | U | L | U | U | U | 3 |
| Decanedioic acid, bis(2-ethylhexyl) ester | 122-62-3 | L-pred | L | L | L | L | L | L | L | L | L | L | L | 3 |
| Diisononyl phthalate | 28553-12-0 | M | L | L-pred | L-pred | L | L | U | U | L | L | L | L | 2 |
| Diisononyl cyclohexane-1,2-dicarboxylate | 166412-78-8 | L | L | L | L | L | L | L | U | L | L | L | L | 4 |
| bis(8-methylnonyl) hexanedioate | 27178-16-1 | L | L | L | L | L | L | U | U | L | L | L | L | 4 |
| Dicylcohexyl phthalate | 84-61-7 | L | L | H | L | L | H | U | L | L | L | L | L | 1 |
| n-butyltri-n-hexyl citrate | 82469-79-2 | M-pred | L-pred | L-pred | L-pred | L | L | L | U | L | L | L | L | 2 |
| tris(6-Methylheptyl)-1,2,4-benzenetricarboxylate | 27251-75-8 | L | L | L | L | L | L | L | U | L | L | L | L | 4 |
| Bis(2-ethylhexyl) cyclohexane-1,2-dicarboxylate | 84-71-9 | M-pred | M-pred | L-pred | L-pred | U | U | U | U | L | U | H | U | 2 |
| Tris(2-ethylhexyl) O-acetylcitrate ATEHC Tributyl O-acetylcitrate | 67989-23-5 | L | L | L | L | L | L | L | L | L | L | L | L | 4 |
| Phthalic anhydride | 85-44-9 | L | L | L | L | L | H | H | U | L | H | vH | M | 2 |
| 1,2,3-Benzotriazole | 95-14-7 | L | L | L | L | L | L | L | U | L | L | H | M | 3 |
| 2-(2-Hydroxyphenyl)benzothiazole | 3411-95-8 | M-pred | M-pred | L-pred | L-pred | M | U | U | U | L | H | H | U | 2 |
| 2-tert-Butyl-6-(5-chloro-2H-benzotriazol-2-yl)-4-methylphenol | 3896-11-5 | L | L | L | L | L | L | U | L | L | L | L | L | 4 |
| 2-Hydroxy-4-n-octoxybenzophenone | 1843-05-6 | M-pred | L | L | L | L | H | U | L | L | L | L | L | 2 |
| 2-(4,6-diphenyl-1 ,3,5-triazin-2-yl)-5-[(hexyl)oxy]-phenol | 25973-55-1 | M-pred | L-pred | L-pred | L-pred | M | L | U | U | L | L | L | L | 2 |
| bis(2,2,6,6-tetramethyl-4-piperidyl)sebacate | 52829-07-9 | L | L | M | L | L | L | U | L | L | L | vH | L | 2 |
| 2,4-Dihydroxybenzophenone | 131-56-6 | M-pred | L | M | L-pred | L | L | U | U | L | L | H | L | 2 |
| 2-(2-Hydroxy-5-tert-octylphenyl)benzotriazole | 3147-75-9 | M-pred | L | L | L | L | L | L | L | L | L | L | L | 2 |
| 2-(2-Hydroxy-3,5-di-tert-amylphenyl)benzotriazole | 70321-86-7 | M-pred | L | L | L | L | L | U | L | L | L | L | L | 2 |
| ethylene thiourea;  Imidazolidine-2-thione | 96-45-7 | H | L | H | H | H | L | U | L | L | L | L | M | 1 |
| Perfluorodecanoic acid | 335-76-2 | M | L | H | H | M | L | L | U | L | H | H | H | 1 |
| 2-Ethyl-1-hexanol | 104-76-7 | L | L | M-pred | M | M | L | L | H | L | H | H | M | 2 |
| 12-Aminododecanolactam | 947-04-6 | L | L | L | L | L | L | U | L | L | L | L | L | 4 |
| Tetradecane | 629-59-4 | L | L | L | L | L | L | L | L | L | L | L | L | 4 |
| 2-Hydroxy-2-methylpropiophenone | 7473-98-5 | M-pred | L | L | L | L | L | U | L | L | L | L | M | 2 |
| 2-Phenyl-2-propanol | 617-94-7 | L-pred | M-pred | L-pred | L-pred | U | L | U | L | L | H | H | M | 2 |
| 1-Dodecene | 112-41-4 | L | L | L | L | L | L | L | L | L | H | L | M | 3 |
| Dodecylbenzenesulfonic acid | 27176-87-0 | L | L | L | L | L | L | L | L | L | vH | vH | M | 2 |
| Didodecyl 3,3'-thiodipropionate | 123-28-4 | L-pred | L | L | L | L | L | U | L | L | L | L | L | 3 |
| Diethylene glycol monoethyl ether | 111-90-0 | L-pred | L | L | L | L | L | U | L | L | L | M | L | 3 |

SnS = skin sensitization; SnR = respiratory sensitization; IrS = skin irritation, IrE = eye irritation. This data was gathered from the sources in Table S8 in the first half of 2024.

# **Table S12.** Analysis of correlation and regression between RRF values and four physicochemical parameters

| **Concentration**  **Parameters** | 5 µg/mL | | 10 µg/mL | | 20 µg/mL | |
| --- | --- | --- | --- | --- | --- | --- |
|  | r | *p* | r | *p* | r | *p* |
|  | **GC-MS** | | | | | |
| **Molecular Weight** | -0.3129 | 0.0078 | -0.2648 | 0.0235 | -0.2795 | 0.0151 |
| **Boiling Point** | -0.4464 | 0.0001 | -0.3403 | 0.0032 | -0.3778 | 0.0008 |
| **LogP** | -0.1072 | 0.3733 | -0.0505 | 0.6709 | -0.0861 | 0.4628 |
| **pKa** | -0.0564 | 0.7002 | -0.0632 | 0.6597 | -0.0868 | 0.5365 |
|  | **LC-MS (positive)** | | | | | |
| **Molecular Weight** | -0.1846 | 0.2303 | -0.1925 | 0.2050 | -0.2116 | 0.1628 |
| **Boiling Point** | -0.2122 | 0.1772 | -0.1963 | 0.2069 | -0.2061 | 0.1848 |
| **LogP** | -0.1820 | 0.2369 | -0.1900 | 0.2111 | -0.2148 | 0.1563 |
| **pKa** | 0.3797 | 0.0320 | 0.3853 | 0.0267 | 0.3692 | 0.0344 |
|  | **LC-MS (negative)** | | | | | |
| **Molecular Weight** | -0.0518 | 0.8945 | -0.0446 | 0.9091 | -0.0362 | 0.9262 |
| **Boiling Point** | -0.2006 | 0.6046 | -0.2118 | 0.5843 | -0.2218 | 0.5661 |
| **LogP** | -0.1019 | 0.7941 | -0.1022 | 0.7936 | -0.0886 | 0.8206 |
| **pKa** | -0.5792 | 0.1021 | -0.5982 | 0.0888 | -0.6136 | 0.0788 |

Note: r: Pearson correlation coefficient *p*: probability that the observed result will occur under the null hypothesis at a significance level of 0.05

# **Table S13.** A comprehensive list of subsets and their corresponding solvents for sample preparation used in GC-MS and LC-MS analyses.

| **Solvent** | **GC-MS** | | **LC-MS, (+)ve** | | **LC-MS, (-)ve** | |
| --- | --- | --- | --- | --- | --- | --- |
|  | **Subset** | **Code** | **Subset** | **Code** | **Subset** | **Code** |
| Methanol | M1-G | RM35, RM40, RM51, RM61, RM63, RM77, RM85, RM87, RM101 | M1-LP | RM35, RM65, RM74,RM77, RM86, RM89,RM99 | M1-LN | RM37, RM46, RM56, RM57, RM62, RM70, RM82, RM84, RM92, RM97, RM104 |
|  | M2-G | RM37, RM48, RM50, RM55, RM66, RM67, RM71, RM72, RM86 | M2-LP | RM40, RM47, RM49,RM63, RM78, RM94 |  |  |
|  | M3-G | RM41, RM52, RM57, RM65, RM74, RM75, RM91, RM97, RM98 | M3-LP | RM34, RM48, RM64, RM72, RM79, RM96, RM105, RM106 |  |  |
|  | M4-G | RM34, RM56, RM60, RM64, RM68, RM79, RM89, RM102, RM105, | M4-LP | RM22, RM43, RM46,RM62, RM82, RM84,RM93, RM104 |  |  |
|  | M5-G | RM33, RM44, RM47, RM49, RM53, RM88, RM93, RM99, RM106 | M5-LP | RM36, RM38, RM39,RM42, RM45, RM54, RM81 |  |  |
|  | M6-G | RM5, RM7, RM10, RM11, RM18, RM19, RM22, RM23, RM70, RM76, RM80, RM92, RM94, RM96 |  |  |  |  |
|  | M7-G | RM1, RM2, RM3, RM4, RM6, RM8, RM9, RM12, RM14, RM15, RM16, RM20 |  |  |  |  |
| Acetonitrile | A1-G | RM13, RM17, RM25, RM26, RM27, RM28, RM29, RM30, RM31, RM32, RM58, RM69, RM90, RM95 | A1-LP | RM1, RM2, RM13,RM14, RM15, RM16,RM17, RM25, RM26,RM27, RM28  RM4, RM5, RM8,RM29, RM30, RM31,RM32, RM56, RM57,RM85, RM93 | A1-LN | RM2, RM4, RM5, RM8, RM13, RM14, RM15, RM16, RM25 RM26, RM27, RM28,RM29, RM30, RM31,RM32, RM56, RM57, RM93 |
|  |  |  |  |  |  |  |
| Hexane | H1-G | RM21, RM24, RM59, RM73, RM83, RM100, RM103 |  |  |  |  |

**References**

1. International Organization for Standardization. ISO 10993-18:2020 Biological evaluation of medical devices - Part 18: Chemical characterization of medical device materials within a risk management process. ISO 10993. Geneva, Switzerland: ISO; 2020.

2. Clean Production Action. GreenScreen® for Safer Chemicals. Clean Production Action: Clean Production Action,; 2019.

3. International Organization for Standardization. ISO/TS 21726:2019 Biological evaluation of medical devices - Application of the threshold of toxicological concern (TTC) for assessing biocompatibility of medical device constituents. ISO/TS 21726 ISO; 2019.

4. Organisation for Economic Co-operation and Development. OECD QSAR Toolbox [Version 5.4]. OECD; 2023.

5. Benfenati E, Manganaro A, Gini G, editors. VEGA in Silico platform -Version 1.2.3. CEUR Workshop Proceedings 2013 2013; Turin, Italy: VEGAHUB.
